# Supplementary material for: Cells Under Stress: An Inertial-Shear Microfluidic Determination of Cell Behavior
Source: Biophys J. 2019 Feb 5;116(6):1127–35. doi: 10.1016/j.bpj.2019.01.034 (PMC6428867; doi:10.1016/j.bpj.2019.01.034)
Supplement: Document S1. Supporting Materials and Methods and Figs. S1–S13 [file mmc1.pdf]

Biophysical Journal, Volume *116*

Supplemental Information

Cells Under Stress: An Inertial-Shear Microfluidic Determination of Cell Behavior

Fern J. Armistead, Julia Gala De Pablo, Hermes Gadêlha, Sally A. Peyman, and Stephen D. Evans

Supporting Information: Cells under stress: An inertial-shear microfluidic determination of cell behaviour

Fern J. Armistead,* Julia Gala De Pablo,* Hermes Gadêlha,[#] Sally A. Peyman,* and Stephen D. Evans*
* Molecular and Nanoscale Physics group, Department of Physics and Astronomy, University of Leeds, Leeds, UK; [#]Department of Mathematics, University of York, York, UK

Reynolds number and drag coefficient calculations:

The Reynold's number Re was calculated for each flow condition, $Re = \frac{2}{3} \cdot \frac{UD_h\rho}{\mu}$, where U is the maximum channel velocity, $D_H = 2wh/(w+h)$ is the hydraulic diameter, w is the channel width, h is the channel height, μ is the dynamic viscosity of the fluid and $\boldsymbol{\rho}$ is the density of the fluid [1]. Supplementary Figure 1 details the relationship between Re and flow rate for a low viscosity (1 cP) and high viscosity (33 cP) of suspension medium.

The compressive force on the cells was calculated using Equation 1, which requires calculation of the Drag Coefficient C_D. The four-parameter drag correlation, $C_D = \frac{24}{Re}(1 + 0.150Re^{0.681}) + \frac{0.407}{(1+\frac{8710}{Re})}$, proposed by Brown *et al.* (2003) was used to calculate the drag coefficient [2], Which is recommended for use when $Re < 2 \cdot 10^5$ which fully encompasses the range of Reynolds numbers used in the body of work. Supplementary Figure 2 further describes the shear and compressive force contributions over a range of flow rates for a low viscosity (1 cP) and high viscosity (33 cP) of suspension medium.

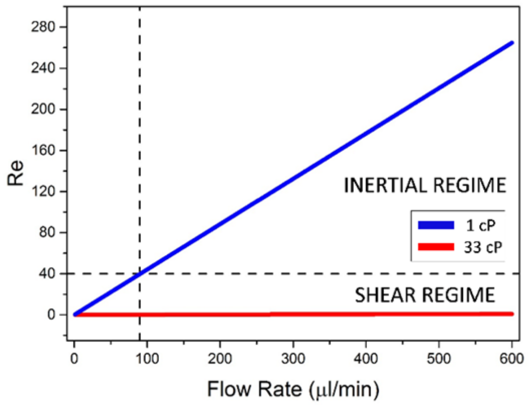

Figure S1: *Variation of Reynolds number with flow rate for our device for two viscosities, μ=1 cP and μ=33 cP. The dashed line at Re=40 represents the flow regime being defined as either shear- or inertia-dominant.*

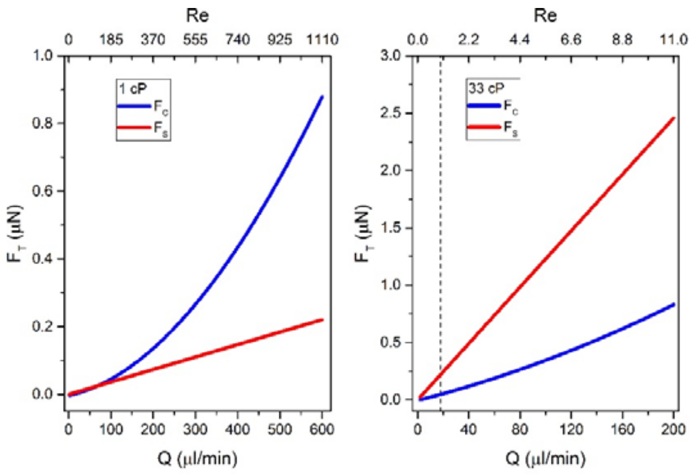

Figure S2: *Plots of equations (1) and (2) as a function of flow rate. Changing the viscosity μ of the fluid determines whether the system is inertia or shear dominant. (a) For μ=1 cP, at flow rates above ~40 μl/min the compressive force contribution F_c begins to surpass the shear contribution F_S. (b) For μ=33 cP the shear force has a larger contribution that the compressive force F_c for the entire range of flow rates described. The*

dashed line is at a Re=1.

Calculation of average DI:

Cell deformation depended on the initial position of cells in the inlet channel. Cells which did not travel centrally down the inlet channel did not deform at or near the SP, and therefore did not undergo the same stresses as a cell deformed at the SP. Thus, these events were excluded from calculations of the DI of each sample. This was done using velocity change of the cell between the inlet channel and the SP. The change in velocity was defined as Δv using equation 1, where v_{inlet} is cell velocity in the inlet channel, and v_{min} is the minimum cell velocity in the cross flow junction. If a cell deforms whilst trapped at the SP v_{min}=0 and ΔV=1. Cells which did not decelerate, and were positioned close to the channel walls, would have Δv=0 because $v_{min}=v_{inlet}$.

Discarding events with Δv<0.75 was chosen as a condition for characterising DI of a sample. Supplementary Figure S3 shows an example dataset of HL60 cells, with the change in average DI shown as a function of Δv threshold, where only cell deformations with Δv greater than the threshold were included in the average. The graph shows a step increase in DI between the thresholds of $0.45 < \Delta v < 0.60$. As the threshold is increased further the DI value plateaus. For Δv>0.8 the standard error begins to significantly increase due to the reduction of events included in the average. Therefore, a threshold of Δv<0.75 was optimum for distinguishing cells deformed close to the SP with a low associated error.

$$\Delta v = \frac{v_{inlet} - v_{min}}{v_{inlet}} \qquad (1)$$

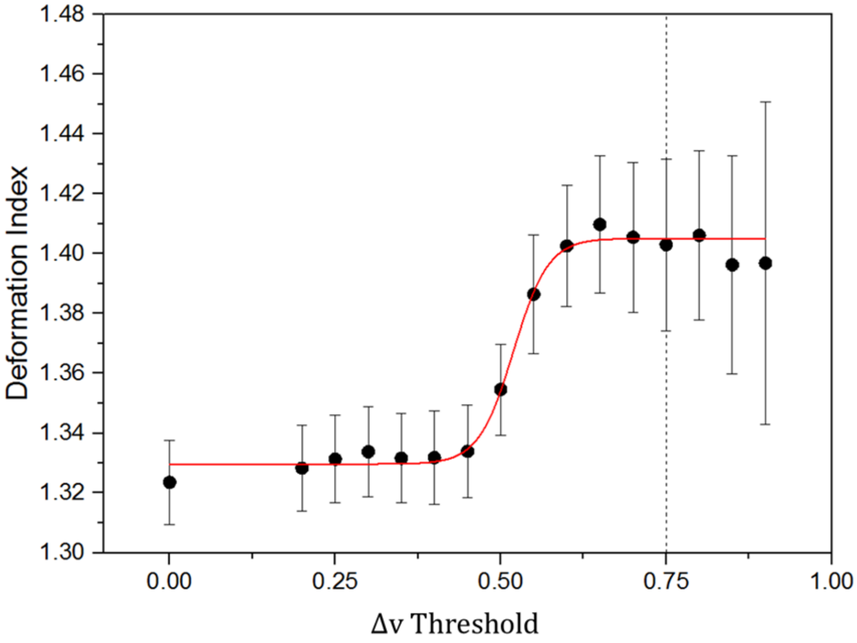

Figure S3: *The average DI±SE of HL60 deformed at a flow rate of 40 µl/min in 0.24% methyl cellulose buffer, as a function of ΔV threshold.*

Velocity profile of microfluidic device:

In this work the velocity profile in the microfluidic device was simulated using the finite element software COMSOL Multiphysics, with the fluid properties $\mu = 33\ cP$ and $\rho = 1005\ kg/m^3$. The simulation was 3D and the geometry mimicked the microfluidic devices used, the channel widths were 35 µm and channel height was 25 µm. A single-phase laminar flow model was used with the initial condition of incompressible fluid behaviour. The boundary conditions were inlet laminar inflow at a flow rate of 5µl/min, and at the outlet pressure of 0. An "extremely fine mesh" was used when running the simulation. Figure S4a shows the variation of flow velocity along the central axis within the cross-flow section of the device, where the position (-40-0) µm is the inlet, position 0 is the stagnation point and (0-40) µm is the outlet. A sine function is fitted to the velocity profile, shown in red.

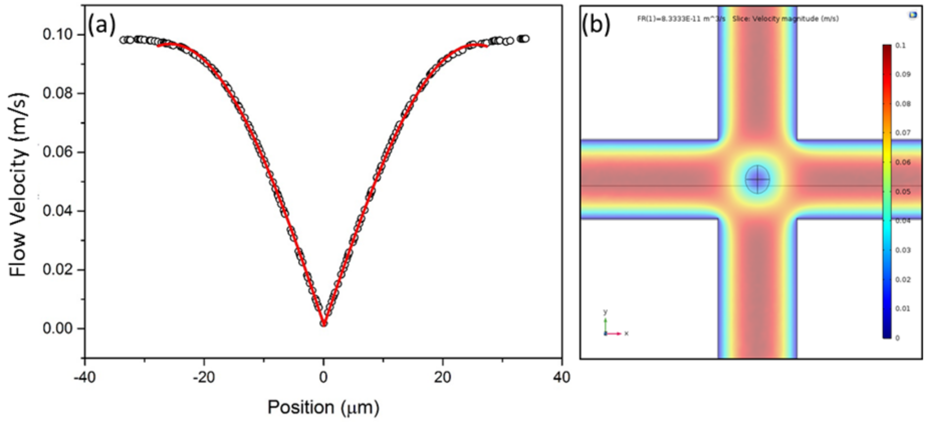

Figure S4: *Velocity profile found using COMSOL where 0 is the stagnation point of the cross-flow. The volumetric flow rate used was 5 µl/min. (b) A velocity magnitude image generated by COMSOL.*

Confocal fluorescence imaging of live SW480 cells treated with Latrunculin A:

SW480 cells detached using TryplE (Thermo Fisher Scientific) and resuspended in DMEM with 0.01 µM, 1 µM and 1 µM of Latrunculin A, as well as a control with no drug added. Cells were incubated with the fluorescent stains and drug for 2 hr before imaging using confocal fluorescence. F-Actin was stained using a live cell fluorogenic labelling probe based on Silicon-Rhodamine (Sir) (Spirochrome, Cytoskeleton Inc.) and DNA was stained using the dye Hoechst 3342 (Thermo Fisher Scientific).

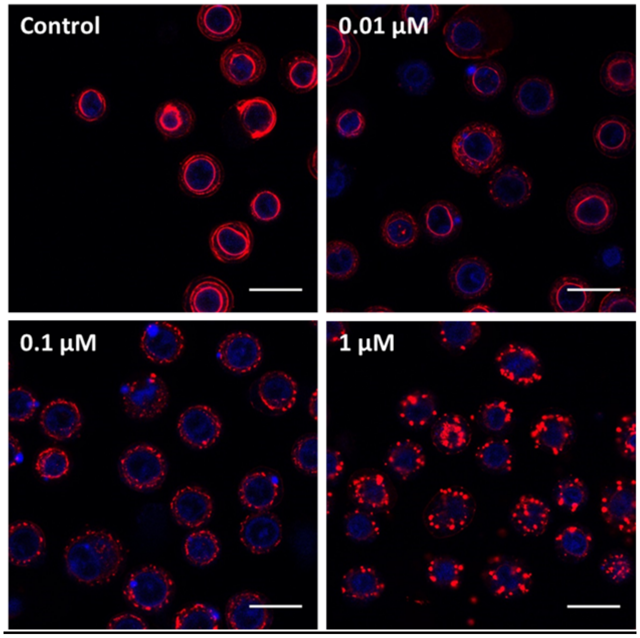

Figure S5: *Confocal fluorescence images of SW480 cells treated with various concentrations of Latrunculin A. Cells were stained for actin (red) and DNA (blue). Images show that with increased concentrations of LatA the actin cortex is less pronounced due to the drug inhibiting actin polymerisation. Thus, the breakdown of the actin causes increased deformability in LatA treated cells. Scale bar 20um.*

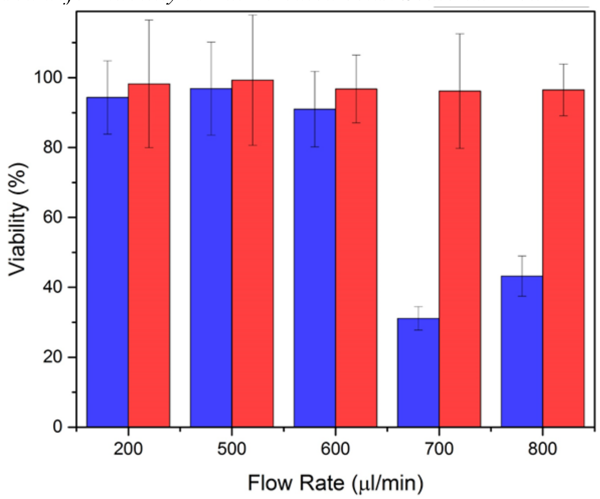

Figure S6: *The viability of HL60 cells after deformation at various flow rates (Q) (blue) in the inertial regime (µ=1 cP), compared to a control which was not deformed (red). Viability assay performed using Trypan blue dye exclusion method*

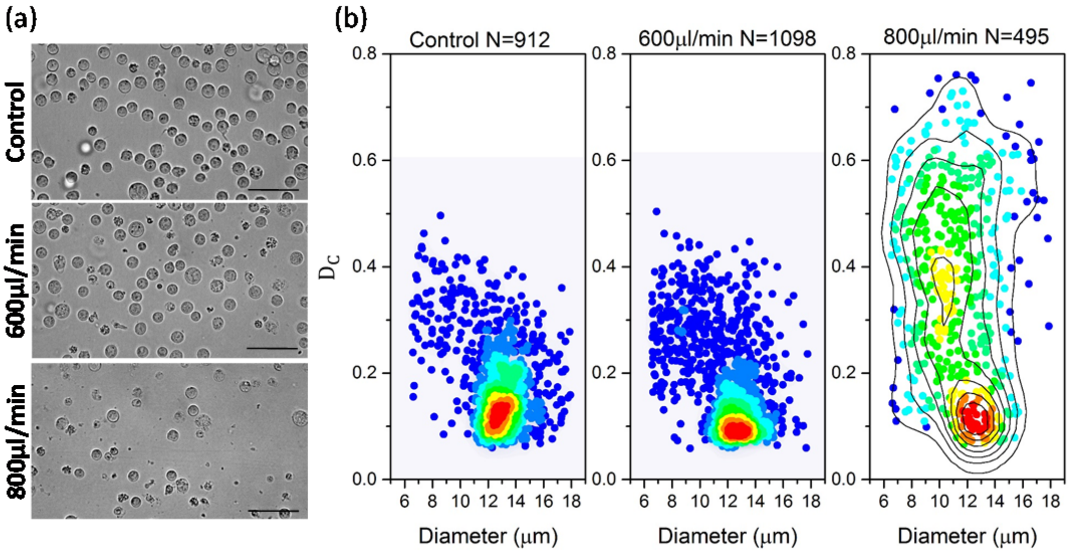

Figure S7: *(a) Phase contrast images of HL60 cells post-deformation in the inertial regime (µ= 1 cP). Scale bar 40 µm. (b) Density scatter plots of HL60 cell shape (D_C) against diameter (µm), comparing cells which were not deformed to those which were deformed at 600 µl/min and 800 µl/min.*

Equation used to determine DI_{max}

Supplementary equation (2) is the exponential fit function used on the datasets in Figure 3. It is a one-phase exponential decay function with constants: amplitude A, time constant τ and offset DI_{max}. This was used to find the extrapolated parameter DI_{max} which represents the maximum deformation the cells plateau towards as a function of flow rate Q.

$$DI = A \cdot e^{-Q\tau} + DI_{max} \quad (2)$$

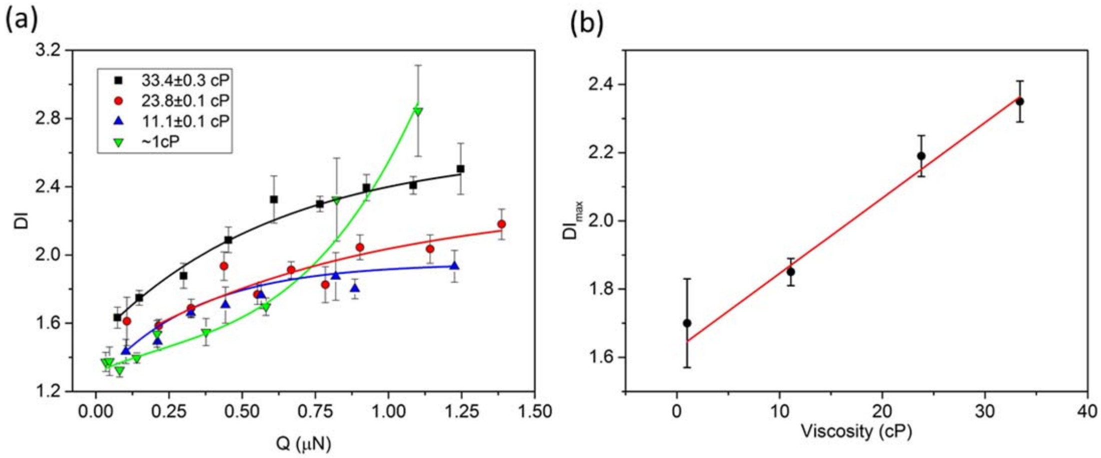

Figure S8: *(a) The maximum deformation of HL60 cells over a range of forces (μN) where* $\boldsymbol{F_T = F_S + F_C}$. *The four data sets represent different flow regimes, where the viscosity of the cell suspension buffer was changed by adding methylcellulose to PBS.* $\boldsymbol{DI \pm SE}$ *was averaged from multiple cell events combined from N=3 repeats, each data point includes 30 > n > 500 cell events. For the same applied force DI was larger in the most shear dominant regime (33 cP) compared to when lower viscosity suspension buffers were used. In the inertial regime (1 cP), for* $\boldsymbol{F_T < 0.58\ \mu N}$ *DI is lower than the shear regime (33cP). For* $\boldsymbol{F_T < 0.58\ \mu N}$ *DI begins to surpass that of the shear regime as this coincides with the cytoskeletal fluidisation regime. The data is fitted with an exponential. (b) A graph of the maximum deformation* $\boldsymbol{D_{max}}$ *of HL60 cells in different viscosity cell suspension mediums, with a linear fit.*

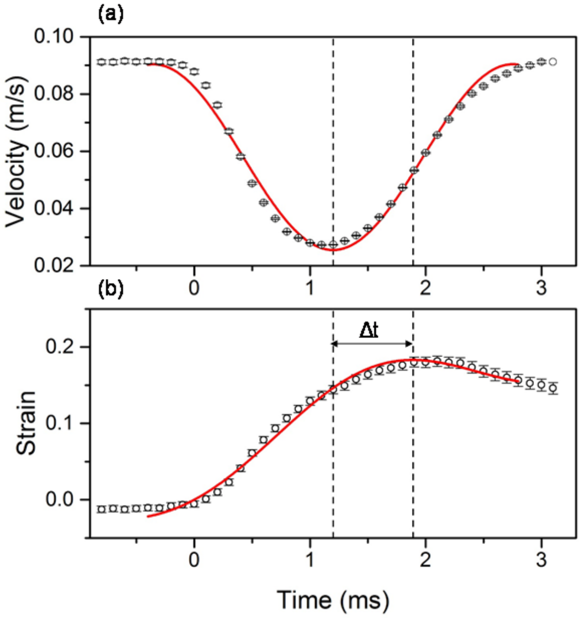

Figure S9: *(a) The average velocity profile of N=50 HL60 cells as they pass through the SP of the cross flow. A sine function is fitted, shown in red. (b) The strain profile of the same 50 cells, the Kelvin-Voigt model was fitted, both shown in red. Q was 5 μl/min and viscosity was μ=33 cP.*

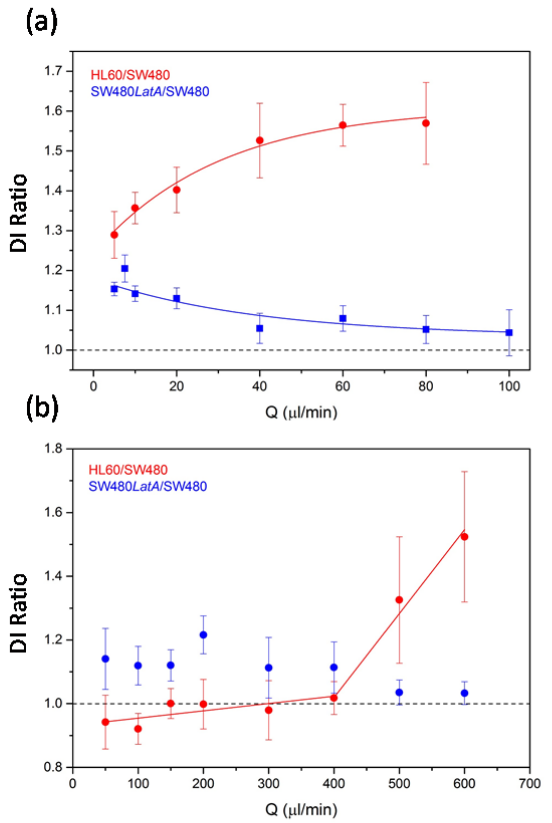

Figure S10: *(a) The DI ratio of the different cell samples as a function of flow rate, in a shear-dominant regime (μ=33 cP). DI ± SE was averaged from multiple cell events combined and from N=3 repeats. The relative DI of HL60 compared to SW480 (DI_{HL60}/DI_{SW480}) is shown in red, the DI ratio of SW480 cells treated with 1 μM of LatA compared to untreated SW480 ($DI_{SW480LatA}/DI_{SW480}$) is shown in blue. Both datasets are fitted with an exponential function. (b) The DI ratio increase of the different cell samples as a function of flow rate, in an inertia-dominant regime (μ~ 1 cP).*

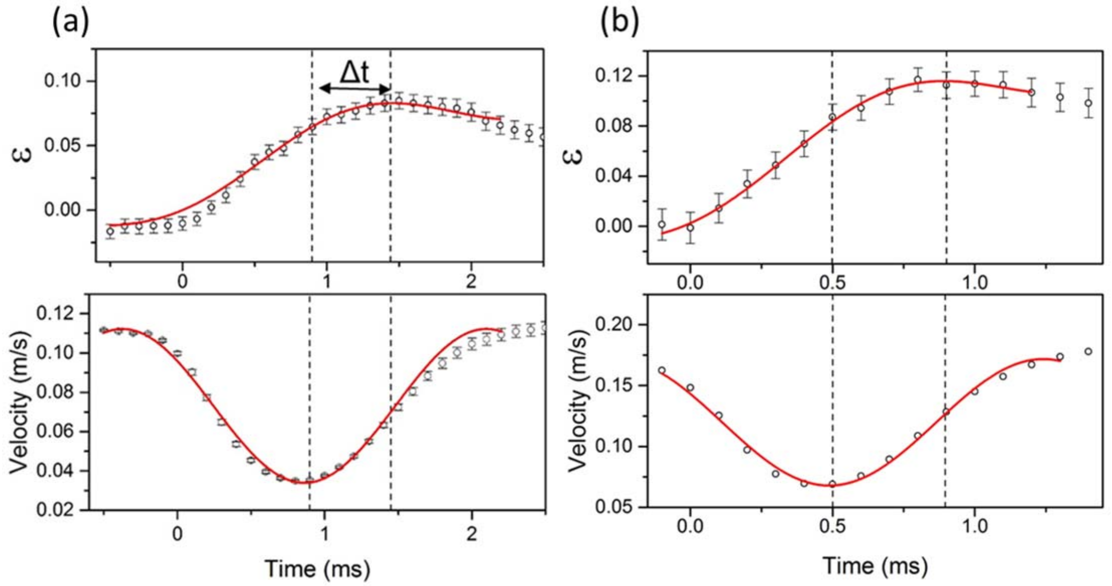

Figure S11: *(a) The strain profile of N=56 SW480 cells, the Kelvin-Voigt model was fitted, shown in red. The average velocity profile of the same 56 cells is shown. A sine function is fitted, shown in red. (b) Strain and velocity profiles for N=30 SW480 cells treated with 1 μM of LatA.*

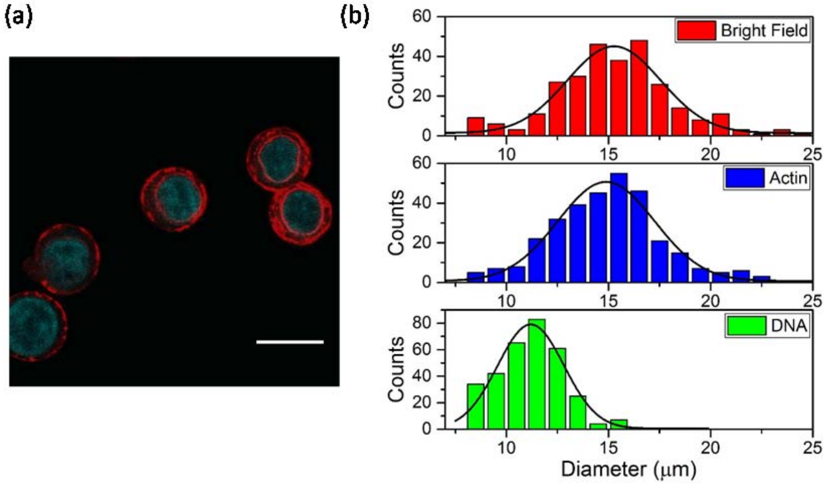

Figure S12: *(a) Confocal fluorescence image of SW480 cells, stained for actin (red) and DNA (blue). (b) Histograms showing the cell diameter found using the bright field image, actin cortex diameter from the actin stain, and the nucleus diameter from the DNA staining.*

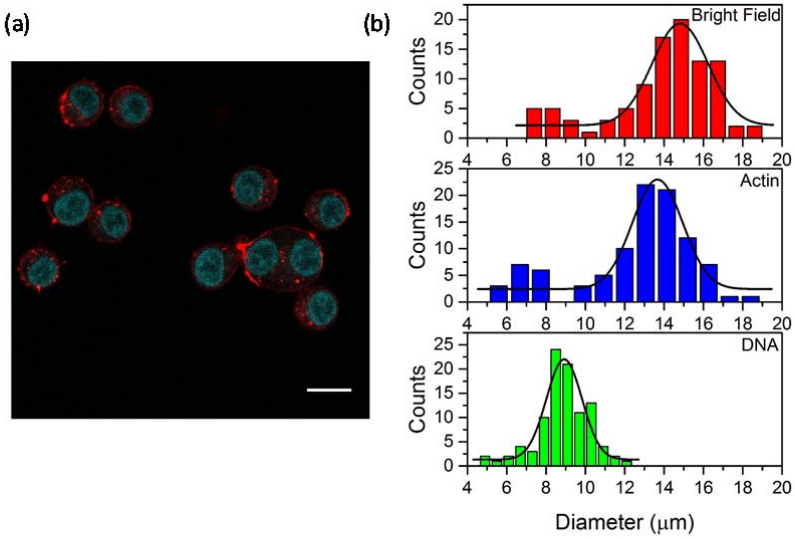

Figure S13: *(a) Confocal fluorescence image of HL60 cells, stained for actin (red) and DNA (blue). (b) Histograms showing the cell diameter found using the bright field image, actin cortex diameter from the actin stain, and the nucleus diameter from the DNA staining.*

Supporting References:

[1] D. Di Carlo, D. Irimia, R. G. Tompkins, and M. Toner, "Continuous inertial focusing, ordering, and separation of particles in microchannels.," *Proc. Natl. Acad. Sci. U. S. A.*, vol. 104, no. 48, pp. 18892–18897, 2007.

[2] P. P. Brown and D. F. Lawler, "Sphere Drag and Settling Velocity Revisited," *J. Environ. Eng.*, vol. 129, no. 3, pp. 222–231, 2003.
