# Supplementary material for: Cells Under Stress: An Inertial-Shear Microfluidic Determination of Cell Behavior
Source: Biophys J. 2019 Feb 5;116(6):1127–35. doi: 10.1016/j.bpj.2019.01.034 (PMC6428867; doi:10.1016/j.bpj.2019.01.034)
Supplement: Document S2. Article plus Supporting Material [file mmc8.pdf]

Biophysical Journal
Article

Biophysical Society

Cells Under Stress: An Inertial-Shear Microfluidic Determination of Cell Behavior

Fern J. Armistead,[1] Julia Gala De Pablo,[1] Hermes Gadêlha,[2] Sally A. Peyman,[1] and Stephen D. Evans[1,*]
[1]Molecular and Nanoscale Physics Group, Department of Physics and Astronomy, University of Leeds, Leeds, United Kingdom and [2]Department of Mathematics, University of York, York, United Kingdom

ABSTRACT The deformability of a cell is the direct result of a complex interplay between the different constituent elements at the subcellular level, coupling a wide range of mechanical responses at different length scales. Changes to the structure of these components can also alter cell phenotype, which points to the critical importance of cell mechanoresponse for diagnostic applications. The response to mechanical stress depends strongly on the forces experienced by the cell. Here, we use cell deformability in both shear-dominant and inertia-dominant microfluidic flow regimes to probe different aspects of the cell structure. In the inertial regime, we follow cellular response from (visco-)elastic through plastic deformation to cell structural failure and show a significant drop in cell viability for shear stresses >11.8 kN/m^2. Comparatively, a shear-dominant regime requires lower applied stresses to achieve higher cell strains. From this regime, deformation traces as a function of time contain a rich source of information including maximal strain, elastic modulus, and cell relaxation times and thus provide a number of markers for distinguishing cell types and potential disease progression. These results emphasize the benefit of multiple parameter determination for improving detection and will ultimately lead to improved accuracy for diagnosis. We present results for leukemia cells (HL60) as a model circulatory cell as well as for a colorectal cancer cell line, SW480, derived from primary adenocarcinoma (Dukes stage B). SW480 were also treated with the actin-disrupting drug latrunculin A to test the sensitivity of flow regimes to the cytoskeleton. We show that the shear regime is more sensitive to cytoskeletal changes and that large strains in the inertial regime cannot resolve changes to the actin cytoskeleton.

INTRODUCTION

Cell deformability is linked to the structure and mechanical properties of its biological constituents, which includes the cytoskeleton, nucleus, and cytoplasm. Disease-induced changes to the cytoskeleton can alter many cellular processes, and cell mechanoresponse is a key biophysical indicator of these changes (1), with distinct mechanical responses being correlated to many diseases (2–5). In recent years, several techniques have been developed to measure cell deformability; these include atomic force microscopy (AFM) (6), optical stretching (7), magnetic twisting cytometry (8,9), micropipette aspiration (3,10), and microfluidics.

Different methods measure localized or whole-cell deformation as well as deforming over different timescales, resulting in widely varying mechanical properties being reported (11,12). Several of these techniques also have limited throughput because the preselection of each single cell is required (6,7,9,10). Mechanical properties can differ on a cell-by-cell basis depending on a cell's physiological state and its stage in the cell-division cycle (13). To determine the heterogeneity of a sample and accurately compare the deformability of different cell types, a high-throughput approach is therefore needed for collection of statistically relevant deformation events. This problem motivated the development of several microfluidics-based methods, which display high-throughput potential ($N > 1000$) and require a small sample volume (13–17).

The mechanical response of cells is affected by the magnitude of the force, the timescale over which the force is applied, and the method of investigation. Previous studies have shown that different microfluidic flow regimes can alter the mechanical response of cells. Gossett et al. (14,17,18) developed the technique deformability cytometry (DC) (19,20) in which cells are hydrodynamically stretched at the stagnation point (SP) of an extensional flow device at rates up to 2000 cells/s. A compressional force (F_C) due to fluid inertia and a shear force (F_S) due to fluid viscosity act on the cells. The F_C contribution was estimated to be ~1000 times greater than F_S, resulting in an inertia-dominant flow regime and high Reynold's number ($Re \gg 1$) (14). Their

Submitted June 11, 2018, and accepted for publication January 30, 2019.
*Correspondence: s.d.evans@leeds.ac.uk
Editor: Jochen Guck.
https://doi.org/10.1016/j.bpj.2019.01.034
© 2019 Biophysical Society.
This is an open access article under the CC BY license (http://creativecommons.org/licenses/by/4.0/).

study showed increased deformability in lymphocyte activation and stem cell pluripotency; these states are characterized by loose, open chromatin structures (18). However, treatment with several cytoskeletal-altering drugs showed negligible changes to cell deformability (14,21). Comparatively, Guillou et al. (22) also used an extensional flow device for single cell deformation but in a regime dominated by shear forces ($Re \ll 1$). They utilized a high-shear, low-velocity, lower-strain regime compared to DC. Here, cells were treated with the actin-disrupting drug cytochalasin D, and they saw an increase in deformability.

Otto et al. (13,23–26) developed real-time DC (RT-DC), which passes cells through a channel slightly larger than the cell, where the strong velocity gradient in the channel causes deformation. RT-DC is also dominated by shear forces ($Re \ll 1$). RT-DC is able to detect deformability changes in cells treated with various cytoskeleton-altering drugs but was not sensitive to changes to the nuclear structure.

It is clear that the sensitivity of deformation cytometry techniques is highly dependent on the flow regime as well as the device geometry, strain, and strain rates applied to the cells. Previous works remain in either a purely shear-dominant or inertia-dominant regime for all studies, and cell deformation is often probed over a small range of flow rates (22,27–30). Here, we deform from low to high strains in both flow regimes, using a single device geometry (Fig. 1 *a*), bridging a critical gap between distant mechanoresponses of the cell.

Microfluidic deformation assays were performed to phenotype two different cell lines. HL60 is a circulating leukemia cell line expected to exhibit a more deformable response compared to SW480 cells, which originate from a solid colorectal cancer tumor. SW480 cells were also treated with an actin-cytoskeleton-disrupting drug, latrunculin A (LatA; Cayman Chemical, Ann Arbor, MI), to determine the sensitivity of the different flow regimes to changes in the actin cytoskeleton. By studying both regimes, we show that specific flow conditions probe different aspects of the cell structure, demonstrating that a shear-dominant and low-strain regime is most sensitive to cytoskeletal changes. Additionally, we found that in the inertial regime, we can achieve a high-strain response resulting in cytoskeletal fluidization and ultimately to failure in the structural integrity of the cell. However, changes caused by LatA could not be resolved in this regime. Viability studies show that cells can remain viable post deformation below the "failure point," meaning the cells could be mechanically phenotyped and continue to be studied.

We also considered which deformation parameters have potential as biophysical markers of the cell's mechanical phenotype. By tracking the deformation and relaxation of the cells, multiple characteristic parameters were extracted, including strain ε, cell velocity profiles, and deformation and relaxation times. The Kelvin-Voigt model was also used to extract an elastic modulus, E, for each cell type, giving us an intrinsic mechanical parameter comparable to previous works using AFM (23,31). Results verified that HL60 are significantly softer than SW480 and that treatment with LatA also reduced the stiffness of SW480. Interestingly, the determination of the different cell types based on relaxation time had the lowest associated error compared with the strain and elastic modulus. These results show the potential of relaxation time as a biophysical marker for mechanical phenotyping and that multiparameter analysis is vital for furthering understanding of cell mechanics.

MATERIALS AND METHODS

Microfluidic devices

Microfluidic devices were fabricated in polydimethylsiloxane (PDMS) using a silicon master as a mold. A silicon wafer (3 inches) was cleaned using

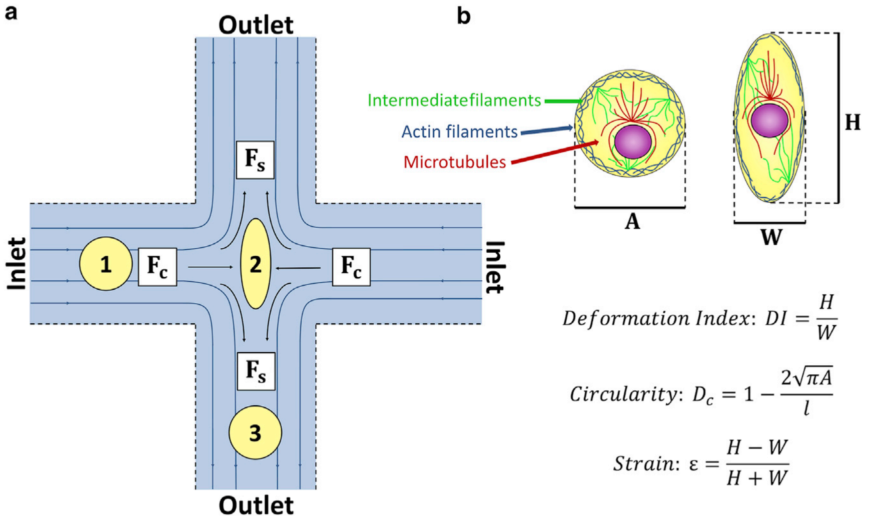

$$Deformation\ Index:\ DI = \frac{H}{W}$$

$$Circularity:\ D_c = 1 - \frac{2\sqrt{\pi A}}{l}$$

$$Strain:\ \varepsilon = \frac{H - W}{H + W}$$

FIGURE 1 (*a*) Schematic of the cross-flow region. (*b*) Parameters extracted from high-speed videos of cell deformation are shown: *A* is the initial diameter of the cell before it deforms, *H* is the height of the cell, *W* is the width of the cell, and *l* is the perimeter of the cell. To see this figure in color, go online.

piranha wet etch (using H_2SO_4 and H_2O_2) and then rinsed with deionized water. The 25-μm photoresist layer of SU-8 2025 (Microchem, Warwickshire, UK) was applied to the wafer. Direct-write laser lithography was used to etch the channel designs into the SU-8 using a laser of wavelength 375 nm (MicroWriter ML; Durham Magneto-Optics, Durham, UK).

A 1:10 ratio of PDMS base and a cross-linking agent (Sylgard 184) were poured onto the master creating a negative replica of the SU-8 structures in PDMS, which was cured in an oven at 75°C for ~1 h, becoming a hydrophobic elastomer. The PDMS layer was then peeled away from the master, and the fluid inlet and outlet access holes are punched using a biopsy puncher. The PDMS was sealed to a glass slide using oxygen plasma treatment. The channel dimensions at the cross-flow junction had a width of 35 μm and a height of 25 μm.

Characterizing flow regime

Cells were deformed at the SP of the extensional flow, defining the deformability using the deformation index $DI = H/W$, where H is the height of the cell and W is the width of the cell (Fig. 1 *b*). The forces acting on a cell can be estimated from the shear and compressive components F_S and F_C. The compressive force F_C was determined from Eq. 1, where ρ is the density of the suspension media, U is the fluid velocity, A_p is the cross-sectional area of the cell. The drag coefficient, C_D, is highly dependent on the Reynolds number, *Re*. The calculation of *Re* and C_D is detailed in Supporting Materials and Methods (32,33). The shear force F_S was determined from Eq. 2, where μ is the viscosity of the suspension media, r is the cell radius, and $\dot{\gamma}$ is the strain rate (14,22). Fig. S1 shows how flow rate and viscosity of the suspension medium can be adjusted to achieve a shear-dominant or inertia-dominant regime. For a solution with a viscosity of 1 centipoise (cP), $Re > 40$ for flow rates ≥ 11 μL/min. Given that inertial effects start for *Re* above 20–40, we use $Re = 40$ as the boundary for the inertial regime (17,34,35). For $\mu = 33$ *cP*, the Reynolds number is low($Re < 6$) and for the entire range of flow rates used in this body of work, which we define as the shear regime.

Fig. S2 further describes the dependence of F_S and F_C as a function of flow rate and the Reynolds number for $\mu = 1$ cP and $\mu = 33$ cP, where the total force F_T is the sum of the two force components, $F_T = F_S + F_C$. F_C increases with density, whereas F_s increases with viscosity. Adding methylcellulose to the suspension buffers led to only a small increase in density but a significant increase in viscosity, resulting in F_T being dominated by F_S. However, F_C increases as U^2 compared to U for F_S. Thus, F_T is dominated by F_C at low viscosity and high flow rates.

$$F_C \cong \frac{1}{2} \rho U^2 C_D A_p \tag{1}$$

$$F_s \cong \dot{\gamma} \mu \left(4 \pi r^2\right) = 2 \pi U \mu r. \tag{2}$$

Experimental procedure

The microfluidic device was mounted above an inverted brightfield microscope (Eclipse Ti-U, Nikon, Tokyo, Japan) with a 10× objective used to capture cell deformation events with an additional 1.5× magnification for flow rates ($Q < 100\mu$L/min). A high-speed camera (Photron, Tokyo, Japan) at a frame rate of 7500–260,000 fps and exposure time of 0.37–6.67 μs was used to capture cell deformation events. An external light source was mounted over the setup to capture images at higher frame rates and reduce exposure times to prevent motion blurring.

Automated image analysis was performed offline using ImageJ and MATLAB, with the position and shape of each cell event tracked and parameters such as initial size, velocity, circularity, and maximal deformation index ($DI = H/W$) extracted. This precision tracking used a mathematical image processing algorithm adapted from flagellar image tracking (36).

Cells that did not travel centrally down the inlet channel and did not deform at the SP were excluded to calculate the average DI of a sample, ensuring all included events experience the same stress during deformation. Methods for calculating DI are described in the Supporting Materials and Methods, including Fig. S3.

Calculation of cell elastic modulus

A Kelvin-Voigt model was fitted to the time-dependent deformation of cells to determine the elastic modulus. This model comprises an elastic element (linear spring) and a viscous element (dashpot) arranged in parallel.

Equation 4 shows the variation of strain rate $\dot{\varepsilon}(t)$ as a function of applied stress $\sigma(t)$, where E is the elastic modulus associated with the linear spring and η is the viscosity associated with the dashpot. In the cross-flow, the stress increases from zero to a maximum of σ_0 as the cell enters the extensional flow junction and reaches the SP. Fig. S4 shows the velocity profile calculated along the central axis within the cross-flow section of the device. This suggests that $\sigma(t)$ varies approximately as a sine function, for a period T. Equation 5 shows $\sigma(t)$, where $\omega = 2\pi/T$, and is used to solve Eq. 4. The analytical solution is shown by Eq. 6, from which the elastic modulus can be directly extracted from the cell deformation dynamics, discussed below.

$$\dot{\varepsilon}(t) = \frac{1}{\eta}(\sigma(t) - E\varepsilon(t)) \tag{4}$$

$$\sigma(t) = \sigma_0(1 + \sin(\omega t)) \tag{5}$$

$$\varepsilon(t) = \frac{\sigma_0}{(\eta^2\omega^2 + E^2)E}\Big(\left(\eta^2\omega^2 - E\eta\omega + E^2\right)e^{-\frac{Et}{\eta}} - E\eta\omega\cos(\omega t) + \omega^2\eta^2 + E^2\sin(\omega t) + E^2\Big). \tag{6}$$

Cell culture

The HL60 cell line was purchased as a frozen stock (ECACC 98070106) and cultured in Roswell Park Memorial Institute growth media supplemented with 10% fetal bovine serum (Sigma, Welwyn Garden City, UK), 2 mM GlutaMax (Thermo Fisher Scientific, Waltham, MA), and penicillin (100 units/mL) and streptomycin (100 μg/mL) (Sigma). HL60 cells are a nonadherent cell line. Centrifuging at 100 × g for 4 min was sufficient to visibly pellet the cells, which were then gently resuspended in the desired suspension medium. Cells were either suspended in Roswell Park Memorial Institute media or resuspended in phosphate-buffered saline (PBS) with 0.24, 0.35, or 0.50% (w/v) methylcellulose (Sigma) to increase viscosity. The viscosity of the cell suspension mediums was measured using a Rheometrics SR-500 Dynamic Stress Rheometer in the parallel plate configuration with a diameter of 25 mm.

The SW480 cell line was provided by St James's University Hospital and cultured in Dulbecco's Modified Eagle Medium (DMEM/F-12; Gibco, Rockville, Maryland) supplemented with 10% fetal bovine serum, 2 mM GlutaMax, and penicillin (100 units/mL) and streptomycin (100 μg/mL). Passage numbers were below 50 for all experiments. SW480 is an adherent cell line and was detached by incubating in TrypLE (Thermo Fisher Scientific) for 5 min. The cells were then centrifuged at 100 × g for 4 min and then resuspended in PBS with 0.5% (w/v) methylcellulose (Sigma).

Drug treatment

SW480 cells were detached by incubation with TrypLE for 5 min, then resuspended in DMEM with varying concentrations of LatA for 2 h. Concentrations of 0.01, 0.1, and 1 μM were compared to a control. Confocal

fluorescence of live cells was used to visualize the effect of LatA on the actin structure of SW480 (Fig. S5). The images show that the actin cortex underwent disruption with increased concentration of LatA because of inhibition of actin polymerization (37).

For subsequent cell deformation experiments, a concentration of 1 μM of LatA was used. Detached cells were incubated with LatA for 2 h before performing microfluidic deformation experiments. Cells were deformed while suspended in DMEM or by resuspension in 0.5% PBS methyl cellulose buffer while maintaining a constant concentration of the drug throughout the measurement period.

RESULTS

Cell deformability in shear and inertial regimes

High-speed imaging was used to capture the maximal deformation of HL60 cells at or near to the SP for a range of flow rates, Q. This was repeated using cell suspension media of increasing viscosity, ranging from 1 to 33 cP, where the 1 cP data set represents an inertia-dominant flow regime, and the 11–33 cP data sets represent an increasingly shear-dominant regime.

Fig. 2 shows deformation in the inertia-dominant regime. For $Q \leq 400$ μL/min, the DI tended toward a plateau, $DI_{\max} = 1.70 \pm 0.13$. For $Q > 400$ μL/min,the DI further increased nonlinearly until $\sim$600 μL/min, beyond which cells rupture and visibly break apart in the cross-flow junction, and deformation could not be measured. The critical deformation DI_{crit} before cell rupture was found to be $DI_{\text{crit}} = 2.84 \pm 0.27$. We can define the stress corresponding to 400 μL/minas being the yield stress of the cell and 600 μL/min as the failure point of the cell. Videos S1, S2, and S3 show example cell deformations occurring at flow rates corresponding below the yield stress at the yield stress and at the failure point. The deformation regime for $Q > 400$ μL/min is associated with the breakdown of the cells internal structure (i.e., actin filament breakup) (38).

Trypan blue staining was used to measure the viability of HL60 cells deformed at various flow rates. The results (Fig. S6) show that cell viability was within the error of the undeformed control for $Q \leq 600$ μL/min. However, for $Q > 600$ μL/min, the viability dropped to <50%. This substantial drop in cell viability occurs at the failure point where cell rupture occurred on-chip. Fig. S7 *a* shows images of samples collected after deformation at 600 and 800 μL/min; for 800 μL/min, there was a reduction the number of viable cells and an associated increase in the amount of debris compared to the control and the 600 μL/min samples. Further, in this regime, the cell did not recover their shape, which was analyzed using the deviation from circularity D_C, defined as $D_C = (1 - \text{c}) = 1 - 2\sqrt{\pi \text{A}}/\ell$, where ℓ is the cell perimeter and c is the circularity; a perfect circle would have $D_C = 0$. Fig. S7 *b* shows low D_C for the control cells (undeformed) and cells deformed at 600 μL/min. For the 800 μL/min values, there is a general increase in the scatter of D_C values resulting from cell debris caused by cell destruction at large flow rates. These results suggest high deformation can be achieved for 400 μL/min < Q < 600 μL/min without adversely affecting cell viability.

Fig. 3 *a* shows DI as a function of Q for solutions of increasing viscosity. For each viscosity, the DI was found to increase asymptotically toward a maximal deformation value $DI_{\max}$, which was determined by fitting an exponential function (see Supporting Materials and Methods). Fig. 3 *b* shows images of cells deformed at $DI_{\max}$ under each flow condition. Each image is accompanied by a superimposed color contour plot, which shows how the deformation varies

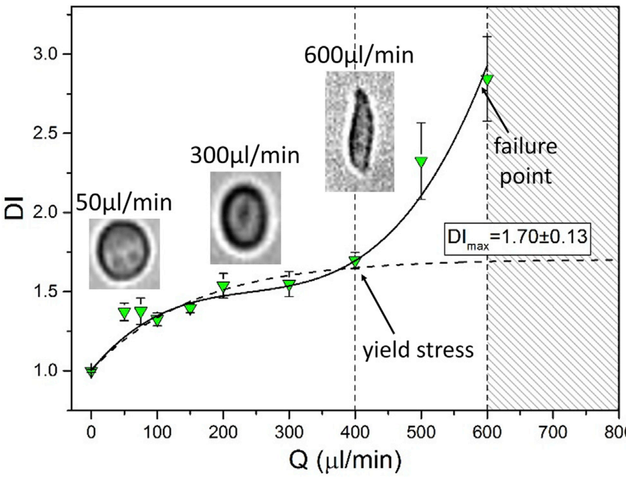

FIGURE 2 Deformation index, DI $\pm$ standard error of HL60 cells versus flow rate, at $\mu = 1$ cP. DI $\pm$ standard error was averaged from multiple cell events combined from $N = 3$ repeats; each data point includes $30 > n > 500$ cell events. For $Q < 400$ μL/min, deformation can be fitted by an exponential, which tends toward a maximal deformation of $DI_{\max}$. To see this figure in color, go online.

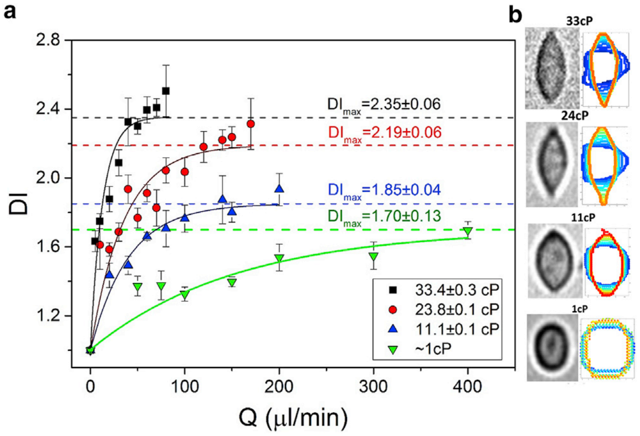

FIGURE 3 (*a*) Deformation index versus Q for HL60 cells in four different media with viscosity changing between 1 and 33 cP. DI $\pm$ standard error was averaged from multiple cell events combined from $N = 3$ repeats; each data point includes $30 > n > 500$ cell events. The data is fitted with an exponential. (*b*) Images of a cell deformation for each flow condition where $DI \cong DI_{\max}$ are shown. They are accompanied by superimposed color contour plots that show how the deformation changes as a function of time. To see this figure in color, go online.

as a function of time, going from blue, where the cell approaches the cross-flow junction, to red, where the cell is deformed at the SP. At higher viscosities, the back pressure in the channels increased, reducing the upper limit of Q achievable without device failure. Despite the limitations in terms of maximal attainable flow rates for the higher viscosity solutions, it is evident that significantly larger deformations can be achieved compared to the inertia-dominant regime. For example, $DI_{max} = 2.35$ for flow rates less than 100 μL/min at 33 cP.

Fig. S8 *a* shows the *DI* data sets plotted as a function of force, F_T, rather than Q. In general, this shows that for the same applied force, the cells were more deformable in a shear-dominant regime. However, this is only true below the previously determined yield stress in the inertia-dominant regime; above this, the *DI* in the inertia-dominant regime exceeds the shear-dominant regime. Additionally, the limiting deformation $DI_{\max}$ varied linearly with viscosity (Fig. S8 *b*).

In addition to measuring *DI*, we also plotted strain (defined as $\boldsymbol{\varepsilon} = \frac{\boldsymbol{H}-\boldsymbol{W}}{\boldsymbol{H}+\boldsymbol{W}}$) versus time to determine the deformation and relaxation times as well as allowing the application of simple models to extract parameters such as elastic modulus *E*.

Fig. 4 *a* shows the average strain of 50 HL60 cells as a function of time, deformed at 5 μL/min in a shear-dominant regime ($\mu = 33.4 \pm 0.3$ cP) (Video S6). A shear-dominant and low-velocity flow condition was chosen to reduce the frame rate (7000 fps) required for tracking and maximize the field of view available. The sign of the strain value describes the cell direction, which changes as the cell moves from the inlet to the outlet, and the magnitude describes the amount of strain. As cells traversed from the inlet to the SP, the strain increased; this was fitted with an exponential with an associated deformation time τ_d.

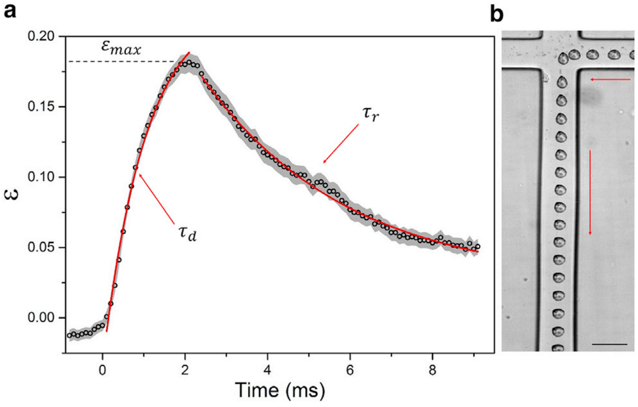

FIGURE 4 (*a*) Strain, ε, as a function of time, averaged over 50 cells, with the standard error shown in gray. Q was fixed at 5 μL/min, and the suspension medium viscosity was 33 cP. The exponential fits shown in red were used to quantify the deformation and relaxation of the cells. (*b*) A superimposed brightfield image of a cell as it deforms and relaxes at 5 μL/min (μ=33 cP) is shown. Scale bars, 30 μm. The arrows indicate the direction of cell motion. To see this figure in color, go online.

Further, it continued to increase as the cell moved from the SP toward the outlet, reaching a maximal strain of $\varepsilon_{\max} = 0.18 \pm 0.04$ (Fig. 4 *b*). The strain then decreased exponentially as the cells traveled toward the outlet and was fitted to an exponential associated with a relaxation time τ_r. Additionally, the initial strain (before entering the SP) ε_0 was found and compared to the final strain value ε_∞, which was found by extrapolation of the exponential fit of the relaxation.

Fig. S9 shows the cell strain and velocity as a function of time. The velocity profile can be roughly approximated to a single period of a sine wave, shown in red in Fig. S9 *a*. The minimum in the velocity profile occurs when the cell is closest to the SP. The sine-oscillating Kelvin-Voigt model (Eq. 5) can then be used to fit the strain trace, shown in red in Fig. S9 *b*.

By equating to the sine-oscillating Kelvin-Voigt model (described in Materials and Methods) the elastic modulus of HL60 was found to be $E = (0.30 \pm 0.03)$ kPa. To demonstrate the potential of this additional parameterization of cell deformation for classifying cell types and understanding disease states, we compared HL60 cells from the circulating leukemia cell line to SW480 cells originating from a primary adenocarcinoma, Dukes stage B.

Fig. 5 *a* shows the *DI* of the cells for a range flow rates up to $\sim$100 μL/min in a shear-dominant regime ($\mu = 33$ cP; Video S4). Firstly, we note that the HL60 cells have significantly a higher *DI* for all flow rates compared to the SW480. Secondly, we observed that treatment of the SW480 cells with the known actin disruptor LatA led to an increase in DI at low flow rates (Fig. S5 shows fluorescence images of LatA-induced disruption of the actin cytoskeleton) (Video S5). In contrast, the *DI* in the inertial regime (Fig. 5 *b*) increases approximately linearly for $Q < 400$ μL/min, with the HL60 cells being slightly more deformable than the SW480 cells. However, on treatment with LatA, the SW480 cells become softer and are similar to the HL60 cells. At $\sim$400 μL/min, the point at which the actin scaffold undergoes significant disruption, there is a change in slope for the HL60 and untreated SW480 cells, whereas the *DI* for the LatA-treated cells continues undeviated. For $Q > 400$ μL/min the LatA-treated cells and untreated SW480 have comparable values of DI.

In the shear-dominant regime, the increase in *DI* of SW480 treated with LatA compared to control cells was more prominent at low flow rates ($Q < 40$ μL/min), with the behavior at higher flow rates asymptotically tending toward untreated behavior. Comparatively, the increase in *DI* of HL60 compared to SW480 increases with flow rate and is more prominent at high flow rates ($Q > 40$ μL/min). In the inertia-dominant regime, there is a small increase in *DI* of SW480 treated with LatA compared to control cells for $Q < 400$ μL/min. The treated and untreated cells are indistinguishable above this flow rate. Contrasting this, HL60 and SW480 have comparable *DI* for $Q < 400$ μL/min; above

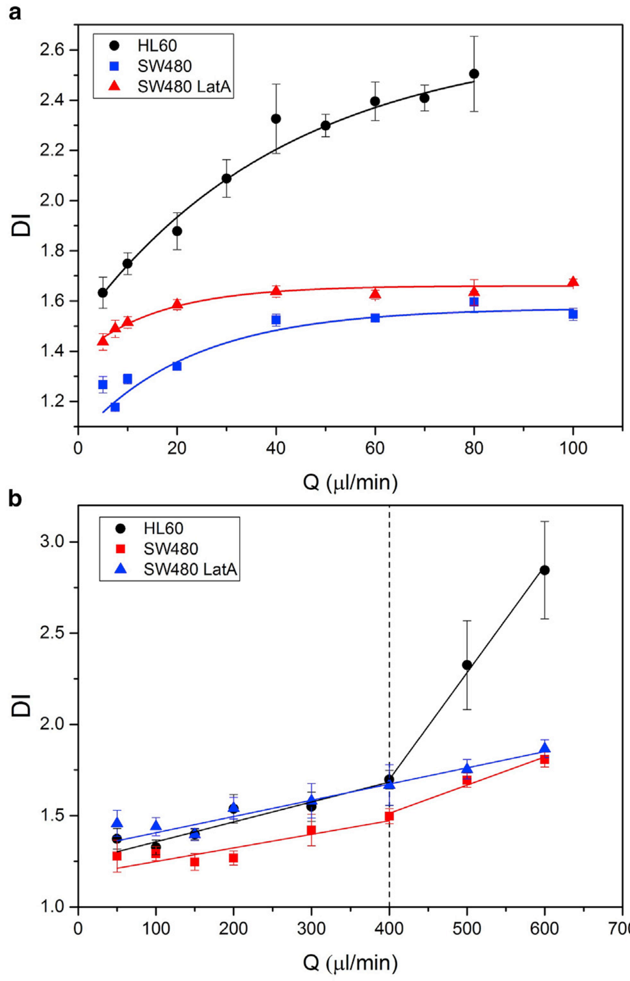

FIGURE 5 (*a*) The *DI* as a function of flow rate, *Q*, of HL60 cells, SW480 cells, and SW480 cells treated with 1 μM of LatA. The flow regime was shear dominant, and the viscosity of the cell suspension buffer was 33 cP. (*b*) *DI* versus flow rate *Q* for HL60 cells, SW480 cells, and SW480 cells treated with 1 μM of LatA. The flow regime was inertia dominant, the viscosity of the cell suspension buffer was ~1 cP. To see this figure in color, go online.

this flow rate, the *DI* of HL60 is significantly higher. Fig. S10 uses the *DI* ratio to show these behaviors explicitly.

Deformation traces for SW480 control and SW480 treated with LatA are shown in Fig. 6, where cells were deformed in a shear-dominant regime ($\mu = 33.4 \pm 0.3\ cP$) at 5 μL/min. Additionally, the velocity and strain profiles are shown in Fig. S11 and were used to fit the Kelvin-Voigt model. The deformation traces show distinct differences, namely, the derived values in the deformation and relaxation times; strain and elastic modulus *E* are shown in Table 1.

DISCUSSION

In the inertia-dominant regime, we identified the point of inflection in the *DI* versus *Q* data (Fig. 2) ($Q > \sim$400 μL/min) with the yield stress for HL60 cells (equating to a force of ~0.58 μN). Below the yield stress, the cells undergo modest changes in deformation, whereas, above the yield stress, the microstructure associated with the actin cytoskeleton breaks down leading to increased deformation (18). Cells that were deformed in this regime are able to recover their original shape and remained viable post-deformation (Fig. S6). However, if cells were deformed beyond the failure point (Fig. 2) ($Q > 600$ μL/min or $DI > 2.84$), then they suffered a significant drop in viability and often did not recover to their original shape post-deformation (Fig. S7). The failure point represents a limit below which live cells can be mechanically phenotyped and collected for further studies, such as chemical phenotyping via Raman spectroscopy (39). For flow rates between 50 and 400 μL/min, only modest changes in *DI* were observed for HL60 cells (1.37–1.70) and similarly for SW480 (1.23–1.47). This regime provides only weak sensitivity to changes in the actin cytoskeleton, as demonstrated by the treatment of

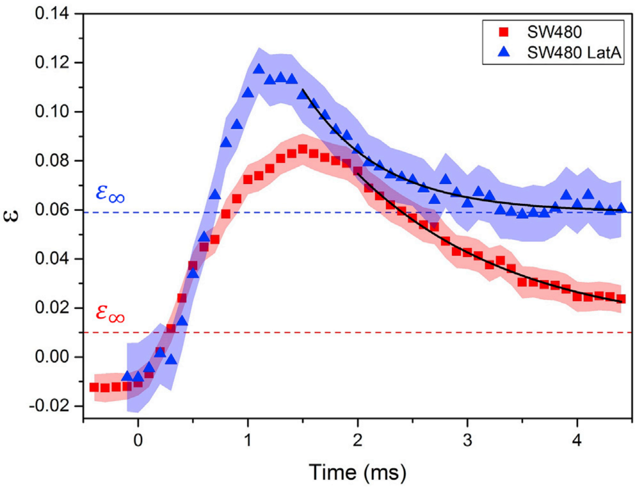

FIGURE 6 Strain ε was tracked for SW480 ($N = 56$) and SW480 treated with LatA ($N = 30$) as a function of time, with the standard error shown. *Q* was fixed at 5 μL/min, and the suspension medium viscosity was 33 cP. The dashed lines represent the extrapolated final strain ε_∞ for both samples. To see this figure in color, go online.

TABLE 1 Multiple Characteristic Parameters Extracted from the Deformation Traces of HL60, SW480, and SW480 LatA Cells, Deforming at the Stagnation Point of an Extensional Flow at 5 μL/min in the Shear Regime

	HL60 ($N = 50$)	SW480 ($N = 56$)	SW480 LatA ($N = 30$)
A (μm)	12.3 $\pm$ 0.3	15.1 $\pm$ 0.2	15.4 $\pm$ 0.1
ε_{max}	0.18 $\pm$ 0.01	0.08 $\pm$ 0.01	0.11 $\pm$ 0.01
τ_r (ms)	3.52 $\pm$ 0.14	1.36 $\pm$ 0.06	0.67 $\pm$ 0.09
τ_d(ms)	1.04 $\pm$ 0.05	1.19 $\pm$ 0.20	0.78 $\pm$ 0.24
$E(Pa)$	301 $\pm$ 29	542 $\pm$ 66	419 $\pm$ 54
ε_0	−0.012 $\pm$ 0.004	−0.012 $\pm$ 0.006	−0.007 $\pm$ 0.014
ε_∞	+0.03 $\pm$ 0.009	+0.010 $\pm$ 0.003	+0.059 $\pm$ 0.001

Where *A* is the cell diameter, ε_{max} is the maximal strain, τ_r is the relaxation time, τ_d is the deformation time, *E* is the elastic modulus, ε_0 is the magnitude of the initial strain, and ε_∞ is the magnitude of the final strain.

the SW480 cells with LatA, after which their *DI* values increased slightly to be similar to those of the intrinsically softer HL60 cells (Fig. 5 *b*). The LatA-treated cells show no obvious change in behavior at $Q \leq \sim 400$ μL/min, whereas the HL60 and untreated SW480 both show increase in gradient of *DI* with *Q*. This observation supports the suggestion that *DI* in the inertial regime is relatively insensitive to the actin scaffold post-degradation of the actin scaffold.

For $Q \leq 400$ μL/min, the HL60 cells show an increase in the slope of *DI* with flow rate compared to SW480 cells; this change is also characterized by the *DI* ratio (Fig. S10 *b*). This could be indicative of the smaller nuclear size in these cells (Figs. S12 and S13). The nuclear ratio ($A_{\text{nucleus}}/A_{\text{cell}}$) of HL60 is 0.55 $\pm$ 0.02 and of SW480 is 0.72 $\pm$ 0.01. The nucleus is known to be significantly stiffer than the surrounding cytoplasm, which is pervaded by the cytoskeleton (40). For example, Caille et al. (41) showed a 10-fold increase in the nucleus elastic moduli of endothelial cells compared to their cytoplasm.

Fig. 3 *a* showed that for HL60 cells, the DI tended toward a maximal value, $DI_{\max}$, with flow rate. The value of $DI_{\max}$ increased linearly with solution viscosity, indicating that at higher viscosities, the same deformation is achieved at lower flow rates (Fig. S7 *b*). It also indicates that inertial and shear forces act very differently on a cell and that for the same magnitude of force, a significantly larger deformation is observed in the shear-dominant regime ($\mu = 33$ cP) (Fig. S8 *a*). Further, the cell shape at maximal deformation changes from elliptical at low viscosity to tear-shapes at higher viscosities, with the cell perimeter changing from convex to concave (Fig. 3 *b*). Deformation in a shear-dominant regime occurs at lower *Re*, and the tear-shape is indicative of the shear force being dominant, causing the pointed ends of the cell.

In the shear-dominant regime as well as in measuring the DI, we captured "deformation traces," which show the variation of cell shape on approach to the SP and relaxation after passing through it (Fig. 4). By plotting these as strain versus time, we obtained a number of parameters related to the cellular state. In particular, the deformation and relaxation times, maximal strain, and through a fit of the Kelvin-Voigt model the elastic modulus. For the HL60 cell line, the elastic modulus, *E*, was found to be 0.30 $\pm$ 0.03 kPa, which falls between those previously reported for HL60 cells obtained from AFM (0.17 $\pm$ 0.03 kPa) by Rosenbluth et al. (31) and RT-DC (1.48 $\pm$ 0.03 kPa) by Mietke et al. (23). Although we would expect our value to be closer to that determined by RT-DC because of the similar timescales of measurement ($\sim$1 ms), we note that RT-DC probes much lower strains and hence potentially different aspects of the cells.

Trikritis et al. (42) used AFM to determine the elastic modulus of SW480 to be 1.39 kPa. Comparatively, Palmieri et al. (43) noted that SW480 cells have two appearances in culture, an epithelial-type morphology and a rounded morphology. Using AFM, they found the elastic modulus of SW480 epithelial-type morphology to be 1.06 kPa and SW480 rounded-type morphology to be 0.58 kPa. The elastic modulus determined here for SW480 rounded-type-morphology cells was within error (i.e., the same as that found by Palmieri et al. (43)). After treatment with LatA, the elastic modulus was reduced to (420 $\pm$ 54) Pa.

Table 1 provides a summary of the multiple parameters extracted from the deformation traces of the different cell types. The values for *E* and ε_{max} confirm that HL60 are significantly softer than SW480 and that treating SW480 with LatA reduced their stiffness. The deformation times τ_d for the three cell types were all within error. However, the relaxation time τ_r can be used to distinguish them from each other. HL60 had the largest relaxation time of $\sim$3.5 ms (which is $\sim$2.5 times longer than for SW480), an expected result considering these are the softest cells. However, the LatA-treated SW480 recover at a faster rate than untreated SW480 despite being more deformable.

The initial strain ε_0 was $\sim$0 for each cell type. The extrapolated final strain ε_∞ was within error of ε_0 for the HL60 and SW480 cell lines ($\varepsilon_0 \cong \varepsilon_\infty$), whereas the LatA-treated cells showed a larger ε_∞ compared to untreated cells ($\varepsilon_0 < \varepsilon_\infty$). Actin disruption using LatA led to a shorter relaxation time being measured, tending to a nonzero strain. This possibly indicates that actin disruption leads to an additional, slower relaxation process occurring over a longer timescale and not fully recovered in our experiments.

CONCLUSIONS

Deformation of HL60 cells as a function of flow rate in the inertia-dominant and shear-dominant regimes show cell response is dependent upon the nature of the applied force (shear, compressive) and not simply the amplitude of the force. Cells appear stiffer in an inertial regime (low viscosity, high flow rate) compared to a shear regime (high viscosity, low flow rate). This behavior indicates that different deformation regimes are likely to be sensitive to different subcellular components. To explore this, we compared two different cell types. The HL60 cells are circulating leukemia cells that exist in the circulatory system, navigating the vasculature and capillary beds as isolated cells. In contrast, the SW480 cells are derived from a primary colorectal adenocarcinoma, a solid tumor. The inertial regime showed several distinct behaviors: at low flow rates, *DI* increased almost linearly with flow rate until the yield point was reached ($Q = \sim 400$ μL/min) at which point the actin scaffold undergoes significant disruption and possible fluidization (38). After this point, 400 μL/min $< Q <$ 600 μL/min the HL60 become significantly more deformable, whereas the change for the SW480 cells is less significant. For these flow rates, the higher stiffness of the SW480 cells is attributed to the larger cell nucleus (and nuclear ratio), being more resistant to deformation. Flow rates above 600 μL/min led to irreversible cell damage with $\sim$50% reduction in cell viability and poor shape recovery post deformation.

In the shear regime, larger DIs were attained with significantly lower flow rates; here, the fluid flow profile probes "stretching deformations" of the cell membrane and the cytoskeleton as opposed to "compressive" behavior of the cytosol and nucleus in the inertial regime. To probe this further, SW480 cells were treated with the actin-disrupting agent LatA. In the shear regime at low flow rates, the LatA-treated SW480 cells were significantly more deformable than the untreated cells; however, as the flow rate was increased, the difference was reduced (see Figs. 5 *a* and S10 *a*), with the SW480 cells approaching that of the actin disrupted cells, thus indicating that the cytoskeleton is probed at the lower flow rates.

As might be expected, the disruption of the actin scaffold using LatA in the inertial regime led to a softening of the cells such that for $Q < 400$ μL/min, the SW480-LatA cells were indistinguishable in their deformability from the HL60 cells. However, at $Q = 400$ μL/min where the actin is disrupted, the HL60 cells become much softer, giving a change in gradient, with a $\Delta DI/\Delta Q$ of $\sim 5.9 \times 10^{-3}$ compared with $\sim 1.1 \times 10^{-3}$ for the lower flow rates. The gradient for the untreated SW480 cells also changes abruptly at this point although with a lower slope. In contrast, the SW480-LatA shows no change in gradient.

In the shear-dominant (cytoskeletal sensitive) regime, we also measured deformation traces and determined multiple characteristic parameters, including maximal strain ε_{max}, initial strain ε_0, final strain ε_∞, elastic modulus E, and relaxation time τ_r. Interestingly, the elastic modulus values of each cell line were of the same order of magnitude as previous AFM measurements despite the different modes and timescales of operation. The fast relaxation of the LatA-treated cells to a nonzero extrapolated final strain suggests that actin disruption causes additional relaxation processes on timescales not recovered in our experiments. Our results show that the multiple parameters have the accuracy to distinguish different cell types and that there is merit in measuring in the shear as well as the inertial regimes to characterize cell response to applied force. The microfluidic approach offers a high-throughput technique for the cell mechanophenotyping as well as increases the range of deformability ($1.3 < DI < 2.8$) and strain rates ($10^3 - 10^5$ Hz) that can be achieved. The data used in the figures of this article will be available at https://doi.org/10.5518/397.

SUPPORTING MATERIAL

Supporting Materials and Methods, thirteen figures, and six videos are available at http://www.biophysj.org/biophysj/supplemental/S0006-3495(19)30104-3.

AUTHOR CONTRIBUTIONS

F.J.A. performed research, analyzed data, and wrote the manuscript. J.G.D.P. helped with culturing of cells and provided code for data analysis. H.G. also provided analytical tools. S.A.P. and S.D.E. helped to design the experimental plan, analyse data, and write the manuscript.

ACKNOWLEDGMENTS

We would like to acknowledge Matthew Hughes for making the viscosity measurements on bulk solutions. We also acknowledge Catriona Marshal (St James's University Hospital) for providing the cell lines and short tandem repeat validation.

F.J.A. thanks the University of Leeds for financial support. S.D.E. acknowledges the following funders: Medical Research Council (MR/M009084/1) and Engineering and Physical Sciences Research Council (EP/P023266/1). S.D.E. is supported by the National Institute for Health Research infrastructure at Leeds. The views expressed are those of the author(s) and not necessarily those of the National Health Service, the National Institute for Health Research, or the Department of Health.

REFERENCES

1. Zheng, Y., J. Nguyen, …, Y. Sun. 2013. Recent advances in microfluidic techniques for single-cell biophysical characterization. *Lab Chip.* 13:2464–2483.
2. Baskurt, O. K., D. Gelmont, and H. J. Meiselman. 1998. Red blood cell deformability in sepsis. *Am. J. Respir. Crit. Care Med.* 157:421–427.
3. Guo, Q., S. J. Reiling, …, H. Ma. 2012. Microfluidic biomechanical assay for red blood cells parasitized by Plasmodium falciparum. *Lab Chip.* 12:1143–1150.
4. McMillan, D. E., N. G. Utterback, and J. La Puma. 1978. Reduced erythrocyte deformability in diabetes. *Diabetes.* 27:895–901.
5. Stuart, J., and G. B. Nash. 1990. Red cell deformability and haematological disorders. *Blood Rev.* 4:141–147.
6. Cross, S. E., Y. S. Jin, …, J. K. Gimzewski. 2007. Nanomechanical analysis of cells from cancer patients. *Nat. Nanotechnol.* 2:780–783.
7. Guck, J., R. Ananthakrishnan, …, J. Käs. 2001. The optical stretcher: a novel laser tool to micromanipulate cells. *Biophys. J.* 81:767–784.
8. Puig-de-morales-marinkovic, M., K. T. Turner, …, S. Suresh. 2007. Viscoelasticity of the human red blood cell. *Am. J. Physiol. Physiol.* 293:C597–C605.
9. Bausch, A. R., F. Ziemann, …, E. Sackmann. 1998. Local measurements of viscoelastic parameters of adherent cell surfaces by magnetic bead microrheometry. *Biophys. J.* 75:2038–2049.
10. Hochmuth, R. M. 2000. Micropipette aspiration of living cells. *J. Biomech.* 33:15–22.
11. Moeendarbary, E., and A. R. Harris. 2014. Cell mechanics: principles, practices, and prospects. *Wiley Interdiscip. Rev. Syst. Biol. Med.* 6:371–388.
12. Suresh, S. 2007. Biomechanics and biophysics of cancer cells. *Acta Biomater.* 3:413–438.
13. Otto, O., P. Rosendahl, …, J. Guck. 2015. Real-time deformability cytometry: on-the-fly cell mechanical phenotyping. *Nat. Methods.* 12:199–202, 4, 202.
14. Gossett, D. R., H. T. Tse, …, D. Di Carlo. 2012. Hydrodynamic stretching of single cells for large population mechanical phenotyping. *Proc. Natl. Acad. Sci. USA.* 109:7630–7635.
15. Forsyth, A. M., J. Wan, …, H. A. Stone. 2010. The dynamic behavior of chemically "stiffened" red blood cells in microchannel flows. *Microvasc. Res.* 80:37–43.
16. Faustino, V., D. Pinho, …, R. Lima. 2014. Extensional flow-based microfluidic device: deformability assessment of red blood cells in contact with tumor cells. *Biochip J.* 8:42–47.
17. Dudani, J. S., D. R. Gossett, …, D. Di Carlo. 2013. Pinched-flow hydrodynamic stretching of single-cells. *Lab Chip.* 13:3728–3734.

18. Tse, H. T., D. R. Gossett, …, D. Di Carlo. 2013. Quantitative diagnosis of malignant pleural effusions by single-cell mechanophenotyping. *Sci. Transl. Med.* 5:212ra163.

19. Che, J., V. Yu, …, D. Di Carlo. 2017. Biophysical isolation and identification of circulating tumor cells. *Lab Chip.* 17:1452–1461.

20. Lin, J., D. Kim, …, D. Di Carlo. 2017. High-throughput physical phenotyping of cell differentiation. *Microsystems and Nanoeng.* 3:17013.

21. Masaeli, M., D. Gupta, …, D. Di Carlo. 2016. Multiparameter mechanical and morphometric screening of cells. *Sci. Rep.* 6:37863.

22. Guillou, L., J. B. Dahl, …, S. J. Muller. 2016. Measuring cell viscoelastic properties using a microfluidic extensional flow device. *Biophys. J.* 111:2039–2050.

23. Mietke, A., O. Otto, …, E. Fischer-Friedrich. 2015. Extracting cell stiffness from real-time deformability cytometry: theory and experiment. *Biophys. J.* 109:2023–2036.

24. Golfier, S., P. Rosendahl, …, O. Otto. 2017. High-throughput cell mechanical phenotyping for label-free titration assays of cytoskeletal modifications. *Cytoskeleton (Hoboken).* 74:283–296.

25. Xavier, M., P. Rosendahl, …, O. Otto. 2016. Mechanical phenotyping of primary human skeletal stem cells in heterogeneous populations by real-time deformability cytometry. *Integr. Biol (Camb).* 16:616–623.

26. Chan, C. J., A. E. Ekpenyong, …, O. Otto. 2015. Myosin II activity softens cells in suspension. *Biophys. J.* 108:1856–1869.

27. Bae, Y. B., H. K. Jang, …, J. M. Kim. 2016. Microfluidic assessment of mechanical cell damage by extensional stress. *Lab Chip.* 16:96–103.

28. Cha, S., T. Shin, …, J. M. Kim. 2012. Cell stretching measurement utilizing viscoelastic particle focusing. *Anal. Chem.* 84:10471–10477.

29. Reymond, L., E. D. Este, …, K. Johnsson. 2014. Fluorogenic probes for live-cell imaging of the cytoskeleton. *Nat. Methods.* 11:731–733.

30. Deng, Y., S. P. Davis, …, A. J. Chung. 2017. Inertial microfluidic cell stretcher (iMCS): fully automated, high-throughput, and near real-time cell mechanotyping. *Small.* 13:1700705.

31. Rosenbluth, M. J., W. A. Lam, and D. A. Fletcher. 2006. Force microscopy of nonadherent cells : a comparison of leukemia cell deformability. *Biophys. J.* 90:2994–3003.

32. Brown, P. P., and D. F. Lawler. 2003. Sphere drag and settling velocity revisited. *J. Environ. Eng.* 129:222–231.

33. Di Carlo, D., D. Irimia, …, M. Toner. 2007. Continuous inertial focusing, ordering, and separation of particles in microchannels. *Proc. Natl. Acad. Sci. USA.* 104:18892–18897.

34. Haward, S. J., T. J. Ober, …, G. H. McKinley. 2012. Extensional rheology and elastic instabilities of a wormlike micellar solution in a microfluidic cross-slot device. *Soft Matter.* 8:536–555.

35. Kim, J. M. 2015. Kinematic analyses of a cross-slot microchannel applicable to cell deformability measurement under inertial or viscoelastic flow. *Korean J. Chem. Eng.* 32:2406–2411.

36. Smith, D. J., E. A. Gaffney, …, J. C. Kirkman-Brown. 2009. Bend propagation in the flagella of migrating human sperm, and Its modulation by viscosity. *Cell Motil. Cytoskeleton.* 66:220–236.

37. Brenner, S. L., and D. Korn. 1987. Inhibition of actin polymerization by latrunculin A. *FEBS Lett.* 2:316–318.

38. Janmey, P. A., U. Euteneuer, …, M. Schliwa. 1991. Viscoelastic properties of vimentin compared with other filamentous biopolymer networks. *J. Cell Biol.* 113:155–160.

39. De Pablo, J. G., M. Lones, …, S. D. Evans. 2018. Biochemical fingerprint of colorectal cancer cell lines using label-free live single-cell Raman spectroscopy 49:1323–1332.

40. Lammerding, J. 2015. Mechanics of the nucleus. *Compr. Physiol.* 1:783–807.

41. Caille, N., O. Thoumine, …, J. Meister. 2002. Contribution of the nucleus to the mechanical properties of endothelial cells. *J. Biomech.* 35:177–187.

42. Trikritis, D., S. Richmond, …, A. Downes. 2015. Label-free identification and characterization of living human primary and secondary tumor cells. *The Analyst.* 140:5162–5168.

43. Palmieri, V., D. Lucchetti, …, M. De Spirito. 2015. Mechanical and structural comparison between primary tumor and lymph node metastasis cells in colorectal cancer. *Soft Matter.* 11:5719–5726.

Biophysical Journal, Volume *116*

Supplemental Information

Cells Under Stress: An Inertial-Shear Microfluidic Determination of Cell Behavior

Fern J. Armistead, Julia Gala De Pablo, Hermes Gadêlha, Sally A. Peyman, and Stephen D. Evans

Supporting Information: Cells under stress: An inertial-shear microfluidic determination of cell behaviour

Fern J. Armistead,* Julia Gala De Pablo,* Hermes Gadêlha,# Sally A. Peyman,* and Stephen D. Evans*

* Molecular and Nanoscale Physics group, Department of Physics and Astronomy, University of Leeds, Leeds, UK; #Department of Mathematics, University of York, York, UK

Reynolds number and drag coefficient calculations:

The Reynold's number Re was calculated for each flow condition, $Re = \frac{2}{3} \cdot \frac{UD_h\rho}{\mu}$, where U is the maximum channel velocity, $D_H = 2wh/(w+h)$ is the hydraulic diameter, w is the channel width, h is the channel height, μ is the dynamic viscosity of the fluid and $\boldsymbol{\rho}$ is the density of the fluid [1]. Supplementary Figure 1 details the relationship between Re and flow rate for a low viscosity (1 cP) and high viscosity (33 cP) of suspension medium.

The compressive force on the cells was calculated using Equation 1, which requires calculation of the Drag Coefficient C_D. The four-parameter drag correlation, $C_D = \frac{24}{Re}(1 + 0.150Re^{0.681}) + \frac{0.407}{(1+\frac{8710}{Re})}$, proposed by Brown *et al.* (2003) was used to calculate the drag coefficient [2], Which is recommended for use when $Re < 2 \cdot 10^5$ which fully encompasses the range of Reynolds numbers used in the body of work. Supplementary Figure 2 further describes the shear and compressive force contributions over a range of flow rates for a low viscosity (1 cP) and high viscosity (33 cP) of suspension medium.

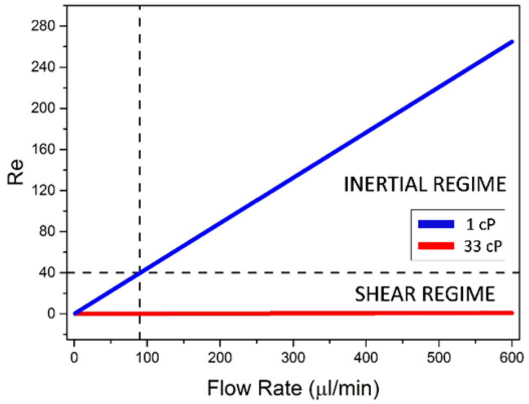

Figure S1: *Variation of Reynolds number with flow rate for our device for two viscosities, μ=1 cP and μ=33 cP. The dashed line at Re=40 represents the flow regime being defined as either shear- or inertia-dominant.*

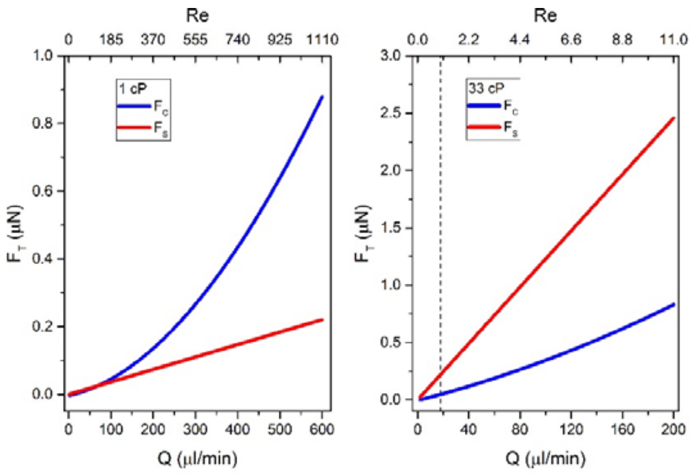

Figure S2: *Plots of equations (1) and (2) as a function of flow rate. Changing the viscosity μ of the fluid determines whether the system is inertia or shear dominant. (a) For μ=1 cP, at flow rates above ~40 μl/min the compressive force contribution F_c begins to surpass the shear contribution F_S. (b) For μ=33 cP the shear force has a larger contribution that the compressive force F_c for the entire range of flow rates described. The*

dashed line is at a Re=1.

Calculation of average DI:

Cell deformation depended on the initial position of cells in the inlet channel. Cells which did not travel centrally down the inlet channel did not deform at or near the SP, and therefore did not undergo the same stresses as a cell deformed at the SP. Thus, these events were excluded from calculations of the DI of each sample. This was done using velocity change of the cell between the inlet channel and the SP. The change in velocity was defined as Δv using equation 1, where v_{inlet} is cell velocity in the inlet channel, and v_{min} is the minimum cell velocity in the cross flow junction. If a cell deforms whilst trapped at the SP v_{min}=0 and ΔV=1. Cells which did not decelerate, and were positioned close to the channel walls, would have Δv=0 because $v_{min}=v_{inlet}$.

Discarding events with Δv<0.75 was chosen as a condition for characterising DI of a sample. Supplementary Figure S3 shows an example dataset of HL60 cells, with the change in average DI shown as a function of Δv threshold, where only cell deformations with Δv greater than the threshold were included in the average. The graph shows a step increase in DI between the thresholds of $0.45 < \Delta v < 0.60$. As the threshold is increased further the DI value plateaus. For Δv>0.8 the standard error begins to significantly increase due to the reduction of events included in the average. Therefore, a threshold of Δv<0.75 was optimum for distinguishing cells deformed close to the SP with a low associated error.

$$\Delta v = \frac{v_{inlet} - v_{min}}{v_{inlet}} \tag{1}$$

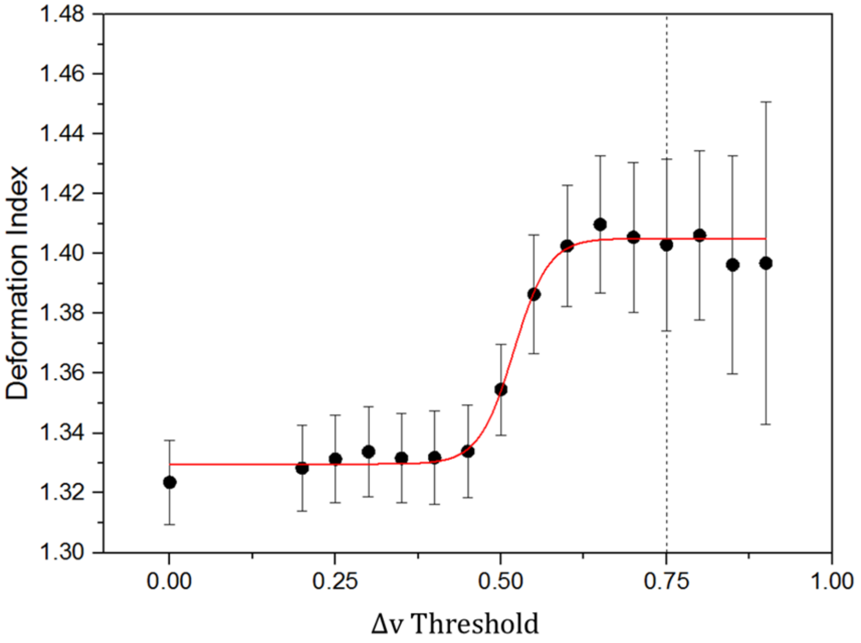

Figure S3: *The average DI±SE of HL60 deformed at a flow rate of 40 µl/min in 0.24% methyl cellulose buffer, as a function of ΔV threshold.*

Velocity profile of microfluidic device:

In this work the velocity profile in the microfluidic device was simulated using the finite element software COMSOL Multiphysics, with the fluid properties $\mu = 33\ cP$ and $\rho = 1005\ kg/m^3$. The simulation was 3D and the geometry mimicked the microfluidic devices used, the channel widths were 35 µm and channel height was 25 µm. A single-phase laminar flow model was used with the initial condition of incompressible fluid behaviour. The boundary conditions were inlet laminar inflow at a flow rate of 5µl/min, and at the outlet pressure of 0. An "extremely fine mesh" was used when running the simulation. Figure S4a shows the variation of flow velocity along the central axis within the cross-flow section of the device, where the position (-40-0) µm is the inlet, position 0 is the stagnation point and (0-40) µm is the outlet. A sine function is fitted to the velocity profile, shown in red.

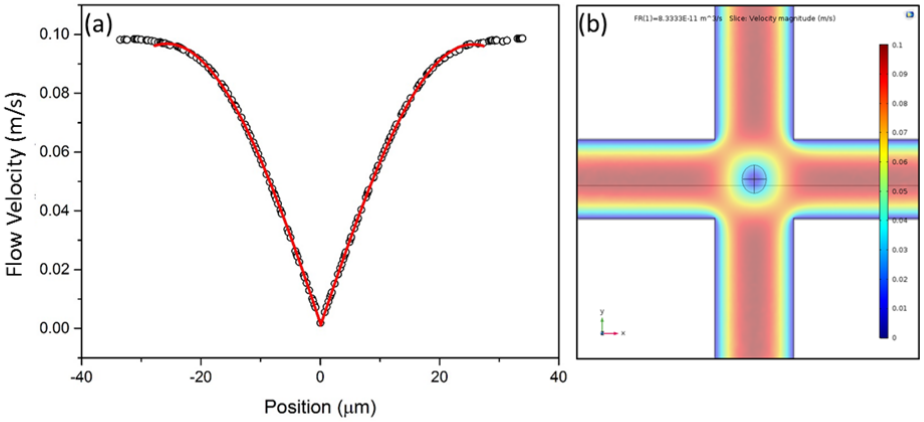

Figure S4: *Velocity profile found using COMSOL where 0 is the stagnation point of the cross-flow. The volumetric flow rate used was 5 µl/min. (b) A velocity magnitude image generated by COMSOL.*

Confocal fluorescence imaging of live SW480 cells treated with Latrunculin A:

SW480 cells detached using TryplE (Thermo Fisher Scientific) and resuspended in DMEM with 0.01 µM, 1 µM and 1 µM of Latrunculin A, as well as a control with no drug added. Cells were incubated with the fluorescent stains and drug for 2 hr before imaging using confocal fluorescence. F-Actin was stained using a live cell fluorogenic labelling probe based on Silicon-Rhodamine (Sir) (Spirochrome, Cytoskeleton Inc.) and DNA was stained using the dye Hoechst 3342 (Thermo Fisher Scientific).

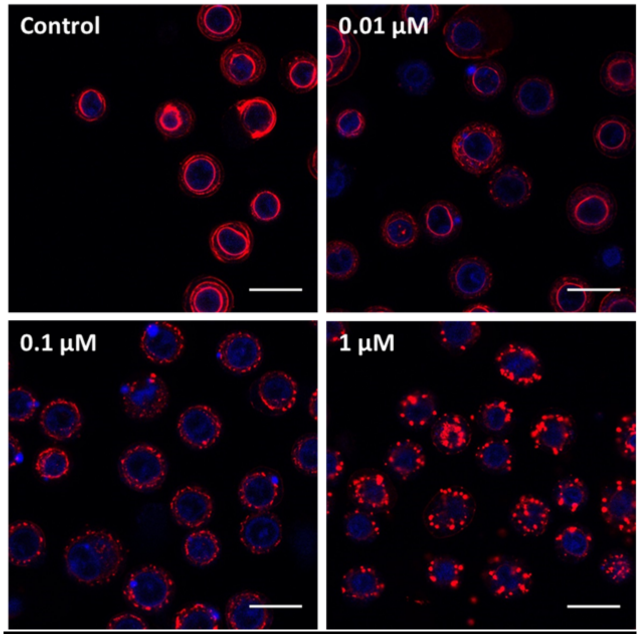

Figure S5: *Confocal fluorescence images of SW480 cells treated with various concentrations of Latrunculin A. Cells were stained for actin (red) and DNA (blue). Images show that with increased concentrations of LatA the actin cortex is less pronounced due to the drug inhibiting actin polymerisation. Thus, the breakdown of the actin causes increased deformability in LatA treated cells. Scale bar 20um.*

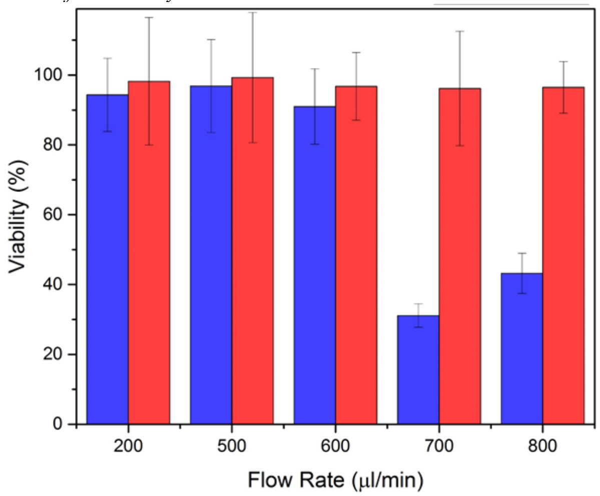

Figure S6: *The viability of HL60 cells after deformation at various flow rates (Q) (blue) in the inertial regime (μ=1 cP), compared to a control which was not deformed (red). Viability assay performed using Trypan blue dye exclusion method*

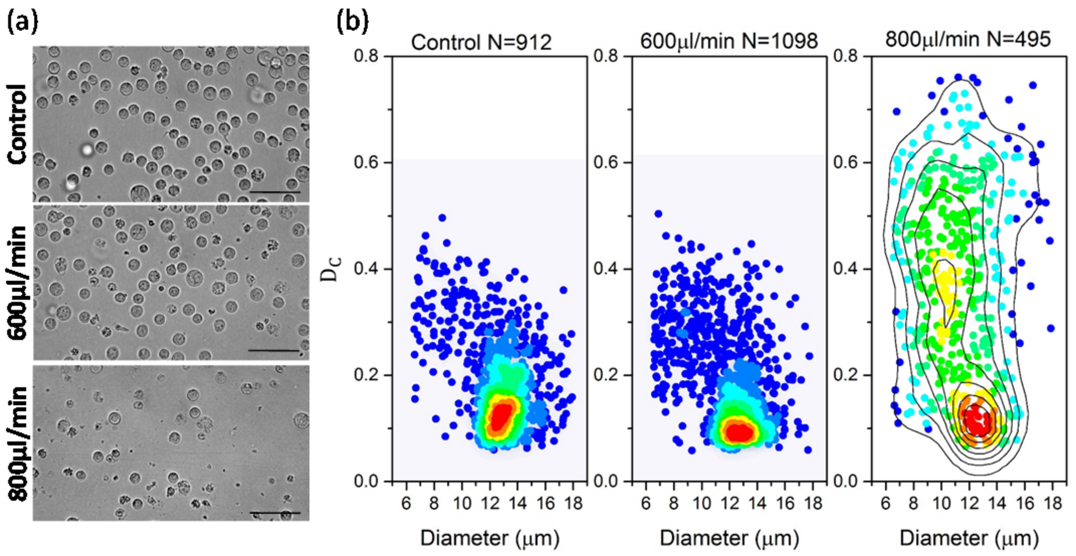

Figure S7: *(a) Phase contrast images of HL60 cells post-deformation in the inertial regime (μ= 1 cP). Scale bar 40 μm. (b) Density scatter plots of HL60 cell shape (D_C) against diameter (μm), comparing cells which were not deformed to those which were deformed at 600 μl/min and 800 μl/min.*

Equation used to determine DI_{max}

Supplementary equation (2) is the exponential fit function used on the datasets in Figure 3. It is a one-phase exponential decay function with constants: amplitude A, time constant τ and offset DI_{max}. This was used to find the extrapolated parameter DI_{max} which represents the maximum deformation the cells plateau towards as a function of flow rate Q.

$$DI = A \cdot e^{-Q\tau} + DI_{max} \tag{2}$$

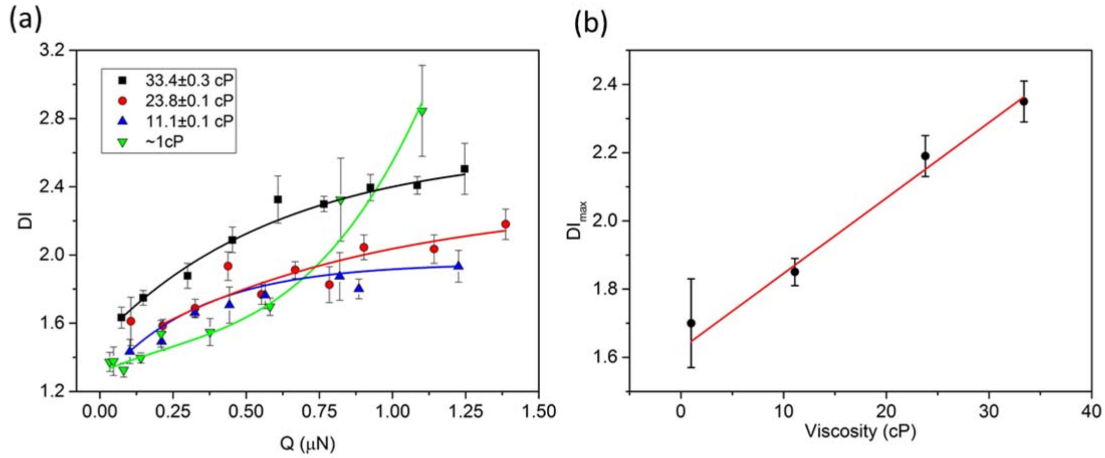

Figure S8: *(a) The maximum deformation of HL60 cells over a range of forces (μN) where $\boldsymbol{F_T = F_S + F_C}$. The four data sets represent different flow regimes, where the viscosity of the cell suspension buffer was changed by adding methylcellulose to PBS. $\boldsymbol{DI \pm SE}$ was averaged from multiple cell events combined from N=3 repeats, each data point includes 30 > n > 500 cell events. For the same applied force DI was larger in the most shear dominant regime (33 cP) compared to when lower viscosity suspension buffers were used. In the inertial regime (1 cP), for $\boldsymbol{F_T < 0.58\ \mu N}$ DI is lower than the shear regime (33cP). For $\boldsymbol{F_T < 0.58\ \mu N}$ DI begins to surpass that of the shear regime as this coincides with the cytoskeletal fluidisation regime. The data is fitted with an exponential. (b) A graph of the maximum deformation $\boldsymbol{D_{max}}$ of HL60 cells in different viscosity cell suspension mediums, with a linear fit.*

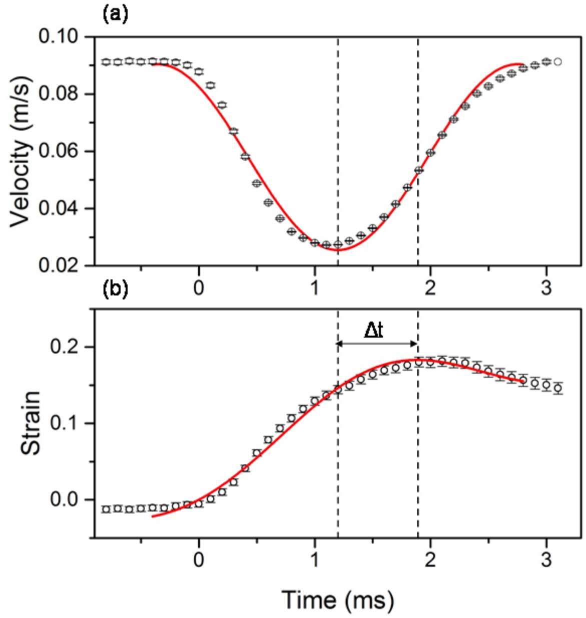

Figure S9: *(a) The average velocity profile of N=50 HL60 cells as they pass through the SP of the cross flow. A sine function is fitted, shown in red. (b) The strain profile of the same 50 cells, the Kelvin-Voigt model was fitted, both shown in red. Q was 5 μl/min and viscosity was μ=33 cP.*

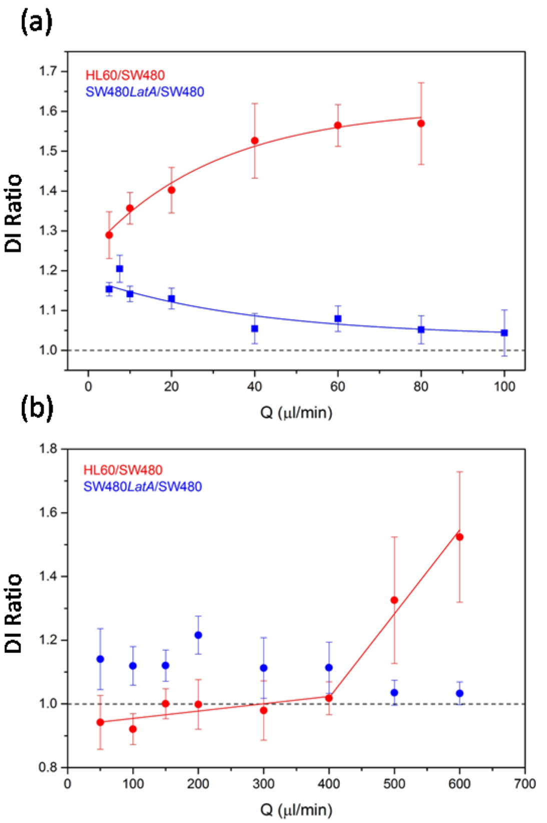

Figure S10: *(a) The DI ratio of the different cell samples as a function of flow rate, in a shear-dominant regime (μ=33 cP). DI* $\pm$ *SE was averaged from multiple cell events combined and from N=3 repeats. The relative DI of HL60 compared to SW480 (*DI_{HL60}/DI_{SW480}*) is shown in red, the DI ratio of SW480 cells treated with 1 μM of LatA compared to untreated SW480 (*$DI_{SW480LatA}/DI_{SW480}$*) is shown in blue. Both datasets are fitted with an exponential function. (b) The DI ratio increase of the different cell samples as a function of flow rate, in an inertia-dominant regime (μ~ 1 cP).*

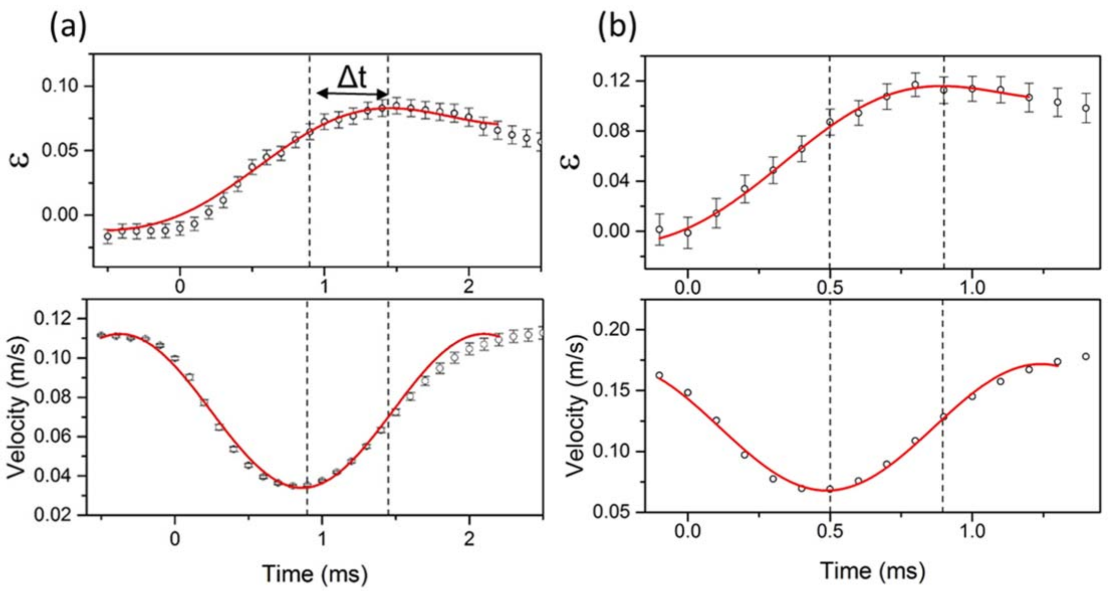

Figure S11: *(a) The strain profile of N=56 SW480 cells, the Kelvin-Voigt model was fitted, shown in red. The average velocity profile of the same 56 cells is shown. A sine function is fitted, shown in red. (b) Strain and velocity profiles for N=30 SW480 cells treated with 1 μM of LatA.*

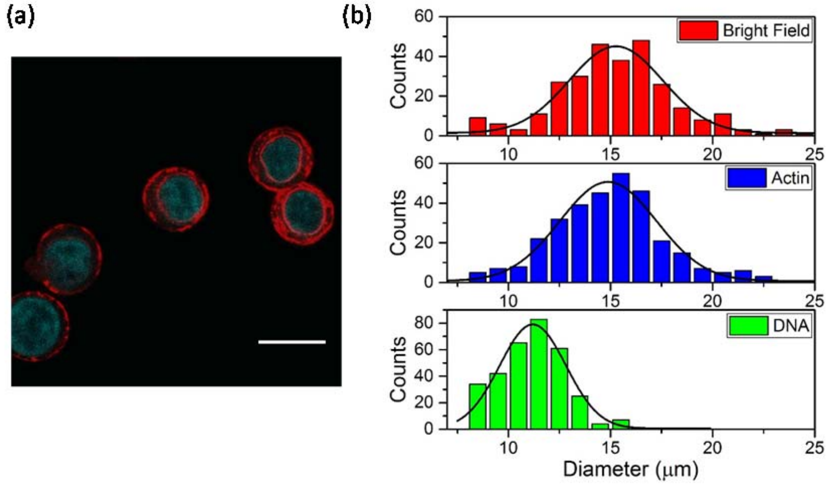

Figure S12: *(a) Confocal fluorescence image of SW480 cells, stained for actin (red) and DNA (blue). (b) Histograms showing the cell diameter found using the bright field image, actin cortex diameter from the actin stain, and the nucleus diameter from the DNA staining.*

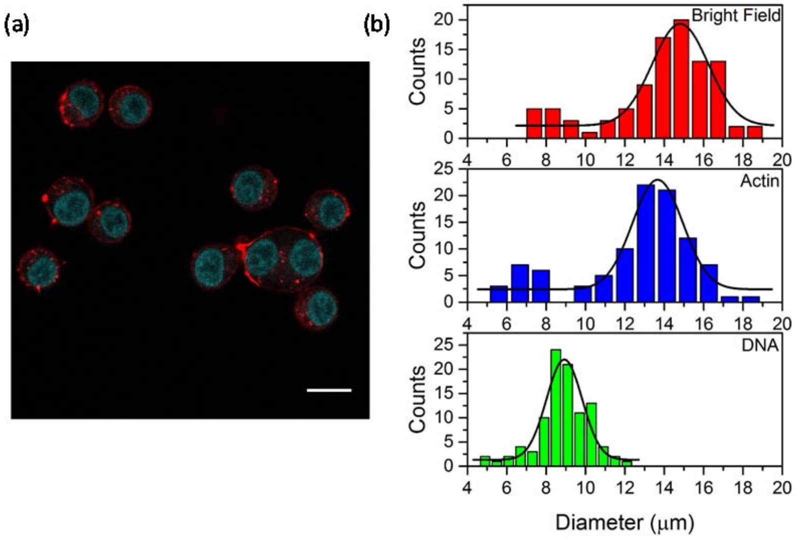

Figure S13: *(a) Confocal fluorescence image of HL60 cells, stained for actin (red) and DNA (blue). (b) Histograms showing the cell diameter found using the bright field image, actin cortex diameter from the actin stain, and the nucleus diameter from the DNA staining.*

Supporting References:

[1] D. Di Carlo, D. Irimia, R. G. Tompkins, and M. Toner, "Continuous inertial focusing, ordering, and separation of particles in microchannels.," *Proc. Natl. Acad. Sci. U. S. A.*, vol. 104, no. 48, pp. 18892–18897, 2007.

[2] P. P. Brown and D. F. Lawler, "Sphere Drag and Settling Velocity Revisited," *J. Environ. Eng.*, vol. 129, no. 3, pp. 222–231, 2003.
